# A global meta-analysis of yield and water productivity responses of vegetables to deficit irrigation

**DOI:** 10.1038/s41598-021-01433-w

**Published:** 2021-11-11

**Authors:** Manpreet Singh, Paramveer Singh, Sukhbir Singh, Rupinder Kaur Saini, Sangamesh V. Angadi

**Affiliations:** 1 Department of Plant and Soil Science, Texas Tech University, Lubbock, TX 79409 USA; 2 Department of Plant and Environmental Sciences, New Mexico State University, Las Cruces, NM 88003 USA

**Keywords:** Plant sciences, Plant stress responses, Drought

## Abstract

Strategies promoting efficient water use and conserving irrigation water are needed to attain water security to meet growing food demands. This meta-analysis study evaluated the effect of deficit irrigation (DI) strategy on eight vegetables to provide a quantitative estimate of yield and water productivity (WP) responses under variable soil textures, climates, and production systems (open-field and greenhouse). This study analyzed 425 yield and 388 WP comparisons of different DI levels to full irrigation (FI), extracted from 185 published studies representing 30 countries. Moving from the highest (> 80%FI) to the lowest (< 35%FI) irrigation level, the overall yield decline was 6.9 to 51.1% compared to FI, respectively. The WP gains ranged from 8.1 to 30.1%, with 35–50%FI recording the highest benefits. Soil texture affected the yield significantly only under the least irrigation class (< 35%FI), wherein sandy clay and loam recorded the highest (82.1%) and the lowest (26.9%) yield decline, respectively. Among the climates, temperate climate was overall the most advantageous with the least yield penalty (21.9%) and the highest WP gain (21.78%) across various DI levels. The DI application under the greenhouse caused lesser yield reduction compared to the open-field. The WP gains due to DI were also higher for greenhouse (18.4%) than open-field (13.6%). Consideration of yield penalties and the cost of saved irrigation water is crucial while devising the reduced irrigation amounts to the crops. The yield reductions under low to moderate water deficits (> 65%FI) accompanied by gains in WP may be justifiable in the light of anticipated water restriction.

## Introduction

Water is a decisive factor for crop production because of its crucial role in nutrient uptake and transport, temperature regulation, and several physiological processes, including photosynthesis. Over the years, water-intensive agriculture has rendered this resource a limiting factor for crop production, especially in water scarce arid and semi-arid areas. Considering the increasing food and nutritional demands of the growing population, a significant part of agricultural research is focused on improving water use efficiency (WUE) and conserving water without yield penalties. Considering the complexity of increasing WUE through breeding owing to the trade-off between photosynthesis and transpiration, agronomic strategies are requisite^1^.

Deficit irrigation (DI) is a direct water conservation approach of reducing water application to improve water productivity (WP). Regulated deficit irrigation (RDI) and partial root zone drying (PRD) are two widely used DI scheduling methods along with the classical DI approach. In RDI, irrigation is either completely withheld or a limited amount of water is applied during a relatively less water sensitive crop growth stage. The PRD alternately exposes the plant roots to wetting and drying cycles in which half root-zone is subjected to water stress at a time. For the sake of uniformity, only the classical DI strategy, in which a percentage of full crop water requirement is applied to the crop during the entire growing season, was selected for this study. The DI approach has been extensively investigated in several crop species, including vegetables, by exposing the crops to different levels of moisture stress^2,3^. Owing to the shallow root systems and sale of vegetable produce on a fresh weight basis, vegetable crops are relatively more sensitive to moisture stress than field crops^3–5^. Consequently, for majority of vegetable crops, DI is often associated with losses in yield and quality^6^. Nevertheless, a small body of evidence indicates that vegetables can adjust to low levels of water deficit producing statistically similar yields as full irrigation (FI)^7–9^. Hence, combination and analysis of yield effects from these studies on a single scale can potentially lead to more robust conclusions. A systematic review comparing the DI effects on yield of various vegetables can reveal the potential of different water stress levels in these vegetable crops and provide a comprehensive picture to the decision makers.

In light of declining water resources around the globe, water restrictions are inevitable. In such a scenario, more emphasis is given to production per unit water use (water productivity) rather than production per unit area (yield). A preliminary review of DI studies revealed that studies rarely focused on a wide range of irrigation levels probably due to funding, labor, area, and time constraints. Additionally, some studies did not provide WP results for which these values had to be calculated. Therefore, statistical pooling of results from these studies is needed to reveal WP effects under a whole range of irrigation levels. A meta-analysis of DI studies reporting WP can also produce important information on threshold DI levels below which WP begins to decline. Previously, researchers have conducted meta-analyses of WP/WUE as affected by irrigation amounts in a number of cereal, fruit, vegetable, and fiber crops at regional^10,11^ and global scales^12–16^. Francaviglia and Di Bene^10^ assessed changes in yield and WP of processing tomatoes under different irrigation classes for the Mediterranean region. A recent global meta-analysis by Cheng, et al.^12^ compared the yield and WP responses of fruits, vegetables (tomato and potato), and field crops under different irrigation regimes without considering the effect of different irrigation levels on individual crops. These previous meta-analyses of vegetables have either evaluated the overall response of vegetables as a group or individually focused on only tomato at a regional scale^10^. A global comparison of the performance of several other important vegetables under different irrigation levels is lacking.

The extent of water stress effects on crop vary with species, genotype, soil characteristics, and climatic conditions. Owing to these factors, discrepancies in yield responses to DI within the same species have been reported^6^. Soil texture is a key soil characteristic that influences plant-water relationships due to its substantial role in determining water infiltration, drainage, hydraulic conductivity, soil water holding capacity, plant available water, and soil aeration^17^. Given this, plants are expected to respond differently to DI under contrasting soil textures. Ahmadi et al.^18^ observed contrasting effects of the same DI level on yield of potatoes grown at three locations with different soil textures. Consequently, soil texture can also influence crop water productivity. Earlier, Martin and Miller^19^ reported similar yield responses under low water deficits but differential responses under high water stress for potatoes grown under sandy and loamy soils. The crop evapotranspiration (ET_c_) demand is determined by climatic factors, such as temperature, radiation, humidity, and wind speed^20^. In arid and semi-arid (dry climate) regions, an additional advection heat increases water loss (both evaporation and transpiration) from the plant-soil system^21^. Even at similar ET-based irrigation rates, crop plants may experience different water stress levels under varied climate. In a multi-location experiment conducted by Bang et al.^22^, 50% ET_c_ restoration reduced the watermelon yield by 36%, 30%, and 15% at three locations with variable weather and soil characteristics. Thus, DI effects on crop yield and WP may vary with the climatic zones. Furthermore, crop responses to DI may vary under greenhouse and open-field conditions due to partial or complete environmental control under greenhouse production. Some studies have compared water use of vegetables in greenhouse vs open-field^23–27^ but rarely evaluated different DI levels simultaneously under two production systems^28^. Since greenhouse production is of utmost importance for vegetable crops, such as tomato, pepper, and cucumber, it is necessary to quantify and compare yield and WP response of vegetable crops under various DI levels in greenhouse and open-field conditions.

The current study employed the meta-analysis method to estimate the vegetable yield and WP responses under various levels of water deficit as affected by variables such as crop species, soil texture, climate, and production system (greenhouse or open-field). Adu et al.^1^ conducted a comprehensive meta-analysis to assess the yield effect size for DI and partial root-zone drying irrigation (PRDI) in comparison to FI for a wide range of crops without considering the differences among levels of water deficit. Zheng et al.^16^ demonstrated the effects of various DI levels on yield and WP of maize (*Zea mays* L.). However, the evaluation of interactive effects of DI levels and the above-mentioned variables is limited. To the best of our knowledge, no such report quantifying yield and WP variation in vegetable crops at different water stress levels is available in the literature. In the present study, we conducted several distinct meta-analyses to illustrate the yield penalties and change in WP associated with different water stress levels and the interactions between water stress levels and the aforementioned candidate variables for vegetable production.

## Results

### Summary of studies

Out of total 331 publications, 185 studies representing eight vegetable crops, including 425 yield comparisons and 388 WP comparisons of different DI levels to FI satisfied the inclusion criteria following the removal of outliers and duplicate studies (Table 1). The time scale of dataset ranged from 1983 to 2021. A study reporting yield results under multiple DI levels provided multiple comparisons, and thus assigned to multiple DI classes. The number of studies in the dataset wes highest on tomato (22%) followed by potato (18%) and onion (14%). Among the DI classes, 50–65%FI and 35–50%FI classes represented the highest and lowest number of comparisons, respectively. The dataset representing 30 countries was dominated by the Middle-East region. The top three countries contributing to the dataset were Turkey (20%), Egypt (12%), and China (10%) (Supplementary Fig. S11). The open-field and greenhouse studies accounted for 78% and 22% of the dataset, respectively (Table 1). A detailed description of the dataset presenting the crop, country, soil texture, climate, and production system for each study is provided as supplementary material (Supplementary Table S1).

**Table 1:** Overview of data included in the meta-analysis.

Crop	Studies	Comparisons (Yield)	Countries	Open-field	Greenhouse
Potato	33	77	14	33	0
Tomato	40	82	16	26	14
Pepper	18	34	11	12	6
Eggplant	20	56	10	15	5
Onion	26	70	12	25	1
Cucumber	20	39	8	9	11
Muskmelon	16	34	8	13	3
Watermelon	14	33	7	14	0
Overall	185	425	30	145	40

### Deficit irrigation effects by crop

Overall, the differences in yield and WP effects among studies were statistically significant (p < 0.05) under all DI classes (Supplementary Fig. S1-S10). The yield effect differences among studies within a crop became more prominent towards lower DI classes (decreasing irrigation rate). For instance, in > 80%FI class, yield differences were significant within tomato (p = 0.00), pepper (p = 0.02), onion (p = 0.00), and watermelon (p = 0.00) subgroups (Supplementary Fig. S1). With slightly lesser irrigation (65–80%FI), the differences were significant within all crop subgroups, except onion (p = 0.07) and muskmelon (p = 0.20) (Supplementary Fig. S2). Further reducing irrigation to 50–60%FI, all subgroup crops but potato (p = 0.10) showed significant differences in yield effects (Supplementary Fig. S3). The WP effects among studies within the crop subgroup also followed a similar pattern (Supplementary Fig. S6-S10).

**Figure 1:**
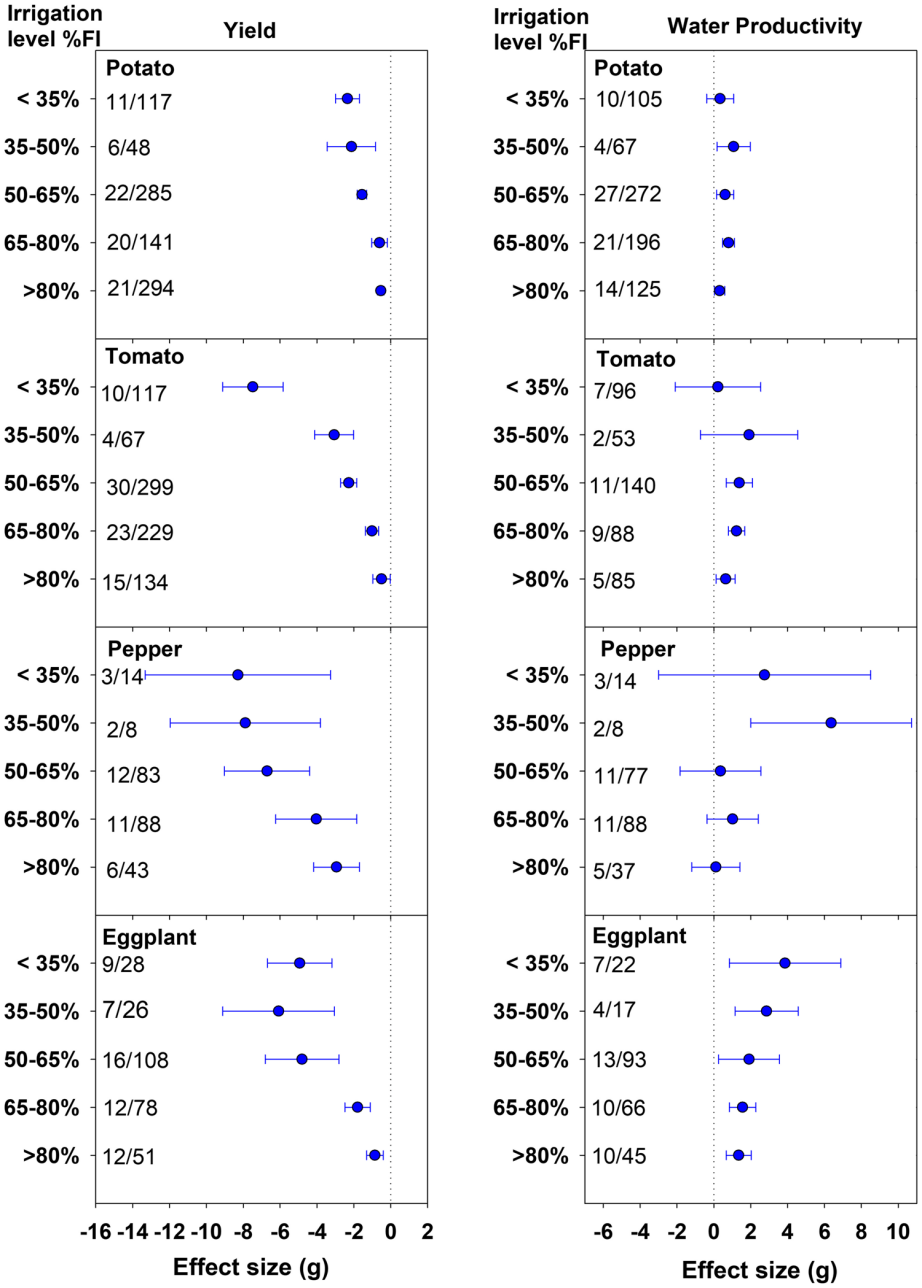
Effect of deficit irrigation levels on yield and water productivity (WP) of potato, tomato, pepper and eggplant. The dotted line (effect size = 0) represents full irrigation. Effect size > 0 indicates increase and effect size < 0 indicates reduction in yield and WP for deficit irrigation level relative to full irrigation (FI). Non-overlapping confidence intervals (horizontal bars) indicate significant differences. Numbers on right side of Y-axis represent number of studies/observations.

**Figure 2:**
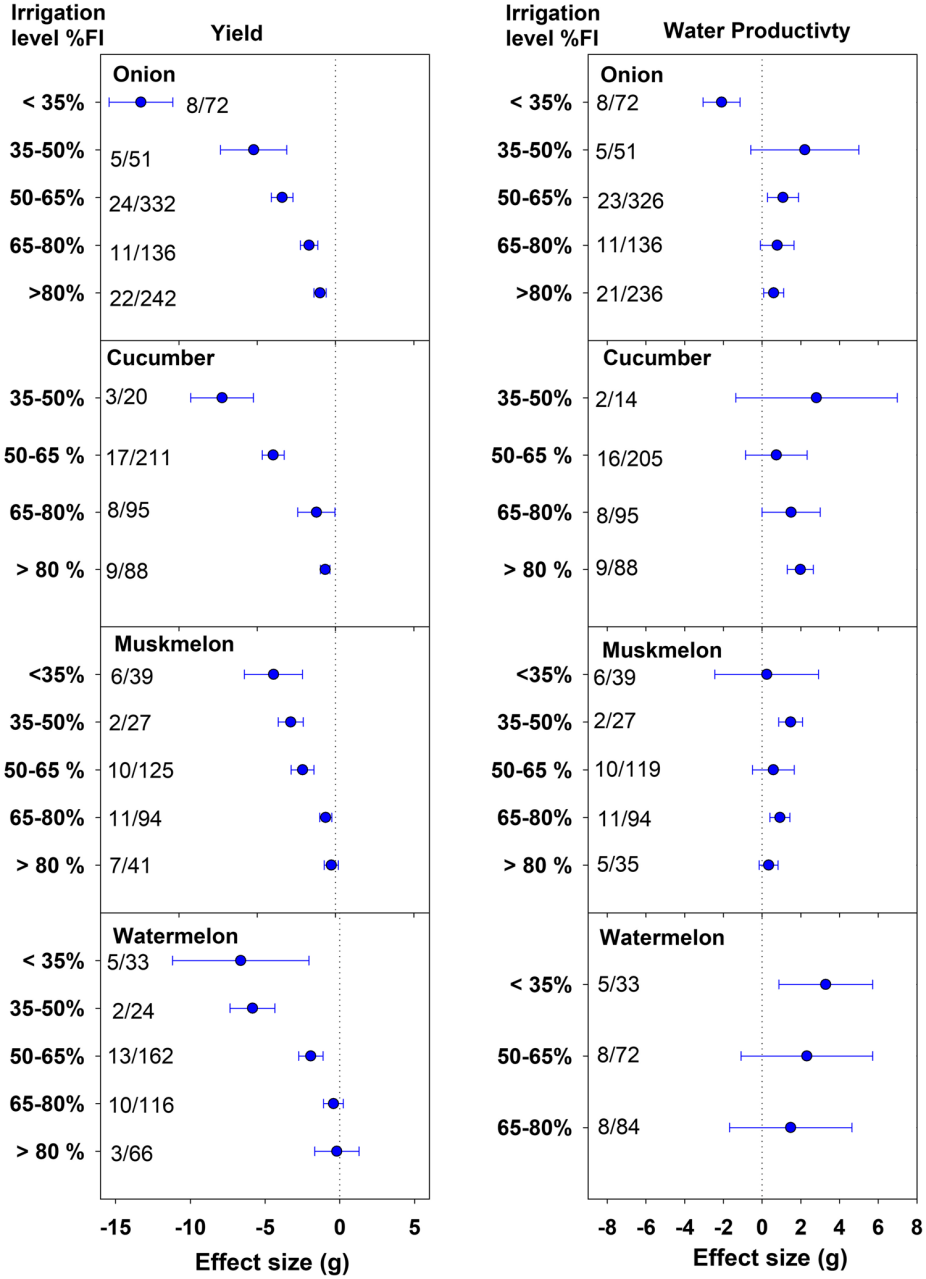
Effect of deficit irrigation levels on yield and water productivity (WP) of onion, cucumber, muskmelon and watermelon. The dotted line (effect size = 0) represents full irrigation. Effect size > 0 indicates increase and effect size < 0 indicates reduction in yield and WP for deficit irrigation level relative to full irrigation (FI). Non-overlapping confidence intervals (horizontal bars) indicate significant differences. Numbers on right side of Y-axis represent number of studies/observations.

All crop species with a few exceptions recorded a significant decline in yield at all DI levels compared to FI (Figs. 1 and 2). Muskmelon and watermelon showed a statistically similar yield as FI up to 80%FI treatment, with only 4.7 and 1.4% yield reduction, respectively (Fig. 2). The yield penalty of 3.9% for watermelon under 65–80%FI, was also non-significant (Figs. 1 and 2). All crop species except pepper recorded less than 10% decline in yield up to 80%FI. As indicated by more negative effect size, the yield penalties increased with increasing water deficit in all crops. In many crops, the yield penalties were moderate up to 35% reduction in irrigation, following a dramatic decline in yield with lower irrigation levels. For instance, in potato the effect size (g) changed from − 0.61 (− 1.04, − 0.18) to − 1.56 (− 1.81, − 1.30) while transitioning from 65–80%FI class to 50–65%FI. Similarly, for tomato, the effect size followed the order of − 0.50, − 1.02, and − 2.28 under 80–95%, 65–80%, and 50–65%FI classes, respectively. In pepper, the yield declined gradually up to 50% water deficit and remained comparable with further reduction in irrigation. Onion observed a low to moderate (8.2 to 25.6%) decline in yield up to 50% water deficit, but a drastic decrease with further reduction in irrigation rate (Fig. 2). Under the least irrigation class, that is < 35%FI, the highest decline in yield was observed in onion [g = − 12.43, CI (− 14.47, − 10.39)] followed by pepper [g = − 8.29, CI (− 13.32, − 3.27)]. For cucumber, < 35%FI class was not included in the meta-analysis due to lack of studies (Fig. 2).

Compared to yield effects, the WP pattern for DI classes was more variable among crop species. Nevertheless, for all crop species, WP either increased significantly or was statistically similar to FI for > 35%FI classes, though the 35–50%FI class for watermelon and < 35%FI for cucumber were not included in the graphs due to lack of studies (Figs. 1 and 2). For solanaceous vegetables, that included potato, tomato, and pepper, the gain in WP (23.2, 33.3, and 44.2%, respectively) was the highest under 35–50%FI class (Fig. 1). Eggplant recorded a significant increase in WP compared to FI under all DI levels. The gains in WP increased with increasing water deficit and peaked at < 35%FI level recording 49.3% gain. Watermelon also recorded the highest WP under the least irrigation class (Fig. 2). In onion, the gain in WP was substantial and statistically significant under 50–65%FI class [g = 1.08, CI (0.28, 1.88)]. The cucumber crop showed a significant gain in WP only under low water deficit class (> 80%FI) with 15.8% increase over FI (Supplementary Table S3). In muskmelon, gain in WP was significant over FI under 35–50%FI and 65–80%FI classes, though the WP results for 35–50%FI in this crop were based only on two studies.

### Deficit irrigation effects by soil texture

**Figure 3:**
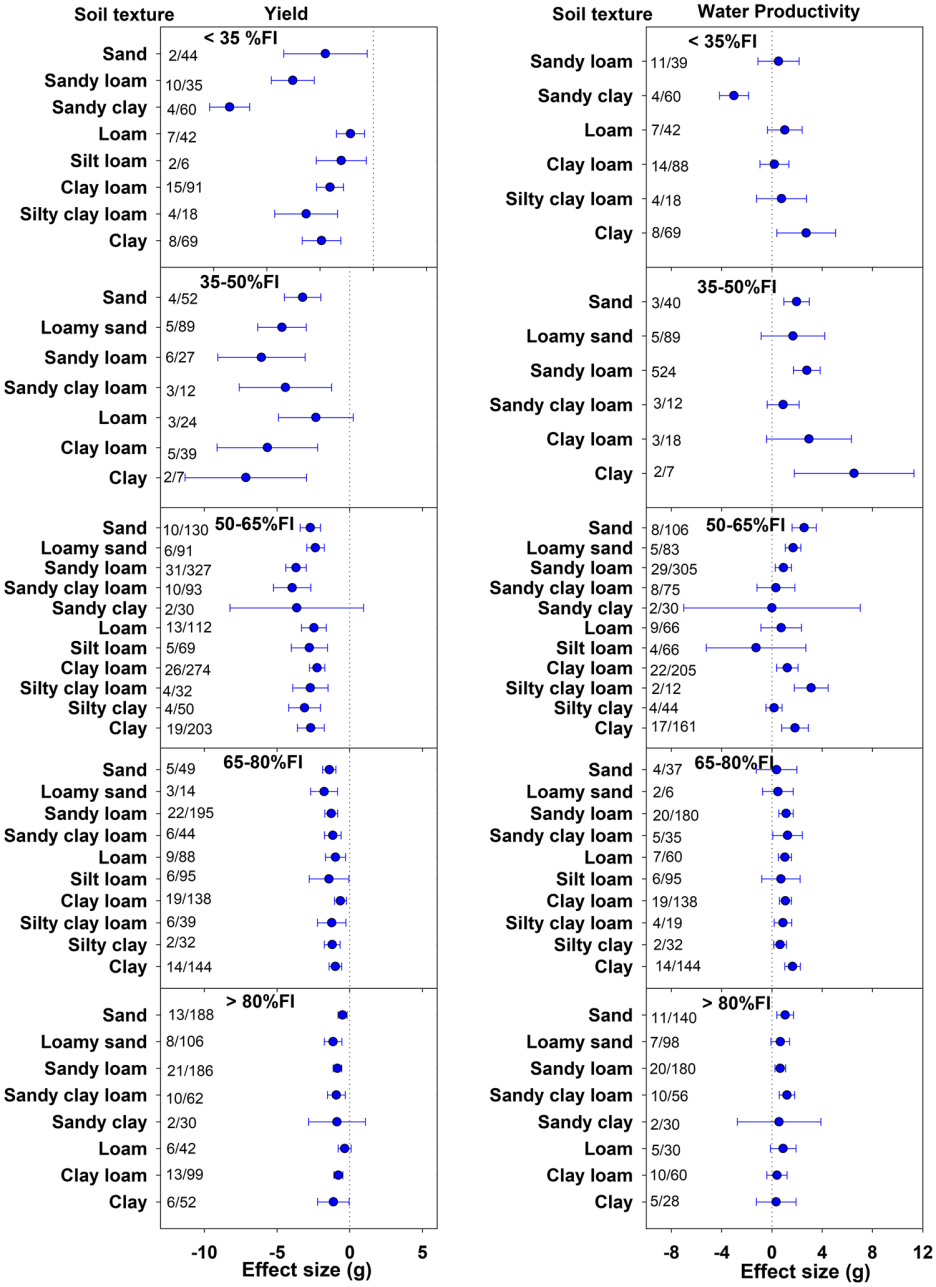
Effect of deficit irrigation levels on yield and water productivity (WP) across different soil textures. The dotted line (effect size = 0) represents full irrigation. Effect size > 0 indicates increase and effect size < 0 indicates reduction in yield and WP for deficit irrigation level relative to full irrigation (FI). Non-overlapping confidence intervals (horizontal bars) indicate significant differences. Numbers on right side of Y-axis represent number of studies/observations.

(Fig. 3). With slightly more irrigation, i.e. at 35–50%FI, although the yield significantly decreased (apart from loam soil), there was a significant increase in WP over FI for some soil textures. The yield was significantly influenced by soil type at < 35%FI. Under this least irrigation class, loam recorded the lowest reduction in yield (26.9%) followed by silt loam (38.2%) and clay loam (44.1%) while sandy clay had highest yield penalty of 82.1%. A similar pattern was observed at 35–50%FI level where loam soil again suffered the lowest yield reduction (28.5%) (Supplementary Table S2). Soil texture did not show a significant influence on yield across 50–65%, 65–80%, and > 80%FI levels, variation in WP due to soil texture was significant under 50–65%FI. At 50–65%FI, silty clay loam, sand, and clay reported the highest and significant increase of 47.2, 28.9, and 26.5% in WP, respectively (Supplementary Table S3). At this irrigation level, only silt loam suffered WP loss of 6.7% compared to FI, though it was not significant. At 65–80%FI, WP was significantly increased in sandy loam, loam, clay loam, and clay soils.

### Deficit irrigation effects by climate

**Figure 4:**
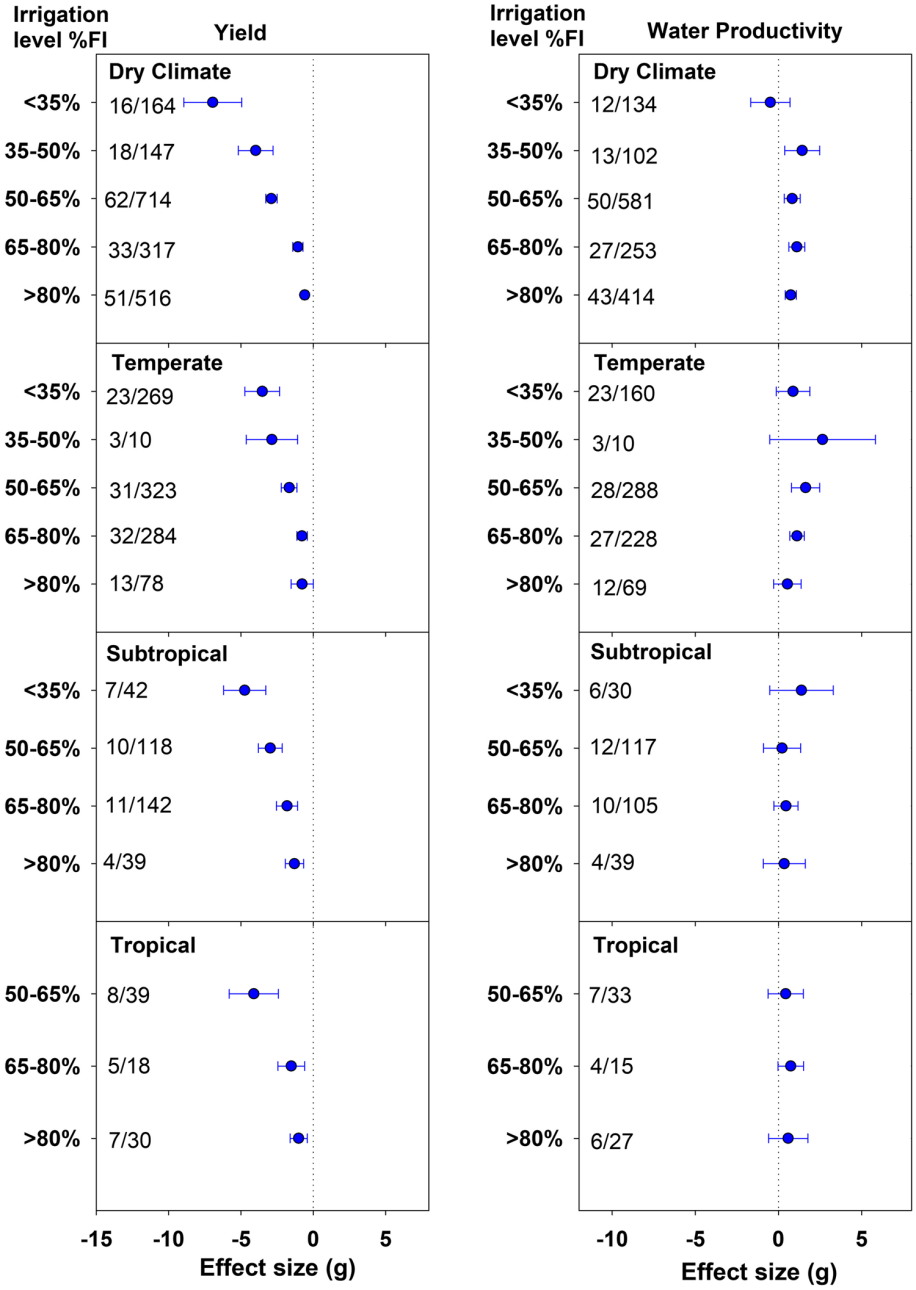
Effect of deficit irrigation levels on yield and water productivity (WP) under different climatic conditions. The dotted line (effect size = 0) represents full irrigation. Effect size > 0 indicates increase and effect size < 0 indicates reduction in yield and WP for deficit irrigation level relative to full irrigation (FI). Non-overlapping confidence intervals (horizontal bars) indicate significant differences. Numbers on right side of Y-axis represent number of studies/observations.

The yield response to various DI levels varied with climatic conditions. Except > 80%FI class, the lowest yield penalties were observed under temperate conditions (Fig. 4.). At 65–80% DI, temperate climate recorded only 6.9% yield decline while the yield reductions in the tropical and subtropical climate were 17.5 and 16.1%, respectively. Although severe yield reductions were observed across all climates at below 65%FI levels, the magnitude varied with climate. For example, under 50–65%FI class, the yield penalty was the highest in the tropical climate (33.9%), while the temperate climate reported the lowest yield reduction (17.5%). The negative effect size obtained at 50–65%FI in tropical was comparable to that obtained under least irrigation (< 35%) in subtropical and temperate climates. At < 35%FI level, dry climate reported the highest yield reduction (64.2%) followed by subtropical climate (41.9%). The 35–50%FI for subtropical, and < 35% and 35–50%FI classes for tropical could not be included due to lack of studies.

Like yield, variation in WP at different DI levels was observed among four climates. In dry and temperate climates, WP increased with a reduction in irrigation application and peaked at 35–50%FI level (Fig. 4). A further reduction in irrigation i.e. < 35%FI resulted in a sharp reduction in WP in both climates. The gains in WP due to DI application were significant only under temperate and dry climates. At 65–80%, DI application increased the WP by 16% in both temperate and dry climates. Reducing the irrigation further to 50–65%FI caused 22.1% gain in WP in temperate compared to 13.4% in dry climate. Under subtropical and tropical climates, WP at all DI levels was at par with FI.

### Open-field vs greenhouse effects

**Figure 5:**
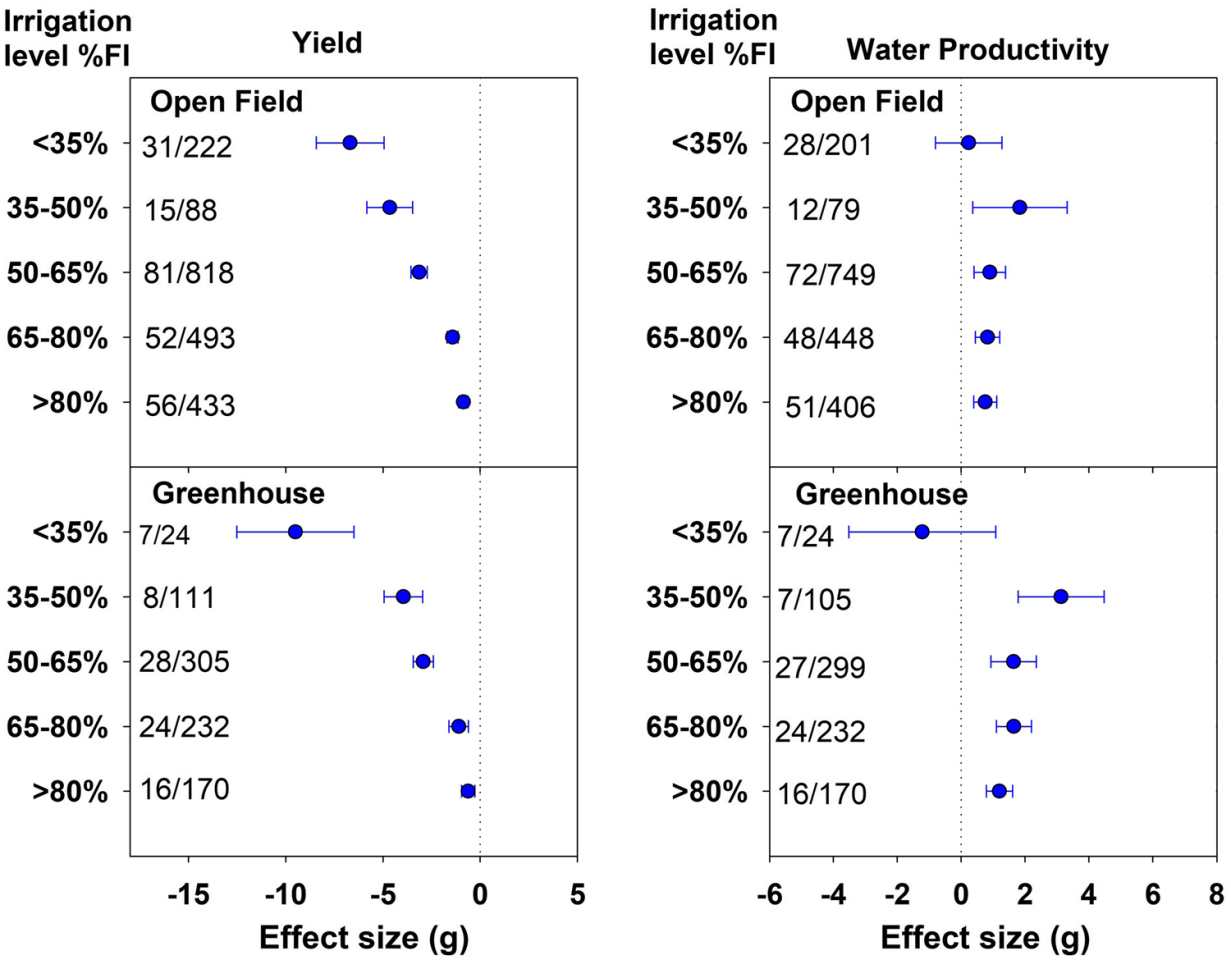
Effect of deficit irrigation levels on yield and water productivity (WP) under open-field and greenhouse conditions. The dotted line (effect size = 0) represents full irrigation. Effect size > 0 indicates increase and effect size < 0 indicates reduction in yield and WP for deficit irrigation level relative to full irrigation (FI). Non-overlapping confidence intervals (horizontal bars) indicate significant differences. Numbers on right side of Y-axis represent number of studies/observations.

The yield effects due to different DI levels followed a similar trend under open-field and greenhouse conditions, in respect of increasing yield penalties with increasing water deficit (Fig. 5). Under both conditions, a dramatic decline in yield was observed transitioning from 65–80%FI to 50–65%FI. The more negative effect size for open-field compared to greenhouse conditions in all DI classes except < 35%FI indicated higher yield penalties under open-field DI application than the greenhouse. Below 35%FI, the greenhouse recorded 77.7% drop in yield which was relatively greater than 57.2% drop observed in open-field.

Under both open-field and greenhouse, the gain in WP increased with increasing water deficit, reaching a maximum at 35–50%FI (Fig. 5). With a further decrease in irrigation, that is, < 35%FI, the WP decreased, the drop being drastic under greenhouse conditions. Under both open-field and greenhouse conditions, the DI application significantly increased the WP compared to FI for irrigation classes with > 35%FI, but the magnitude of WP gains was much higher in greenhouse than open-field. Excluding the < 35%FI class, DI application resulted in average WP gain of 24.5% under greenhouse compared to 14% under open-field conditions.

## Discussion

The magnitude of yield reduction increased with increasing water deficit. Analysis of effect size revealed a significant decline in yield for all DI classes in comparison to FI for all crops with a few exceptions confirming to the fact that a majority of vegetables are sensitive to water stress. However, relatively smaller yield reductions under low to moderate water deficits (> 65%FI) can be acceptable in areas with diminishing water resources, especially for muskmelon and watermelon, which produced statistically similar yield as FI under low water deficits. Besides, the yield penalties under these DI levels may be compensated by an increase in WP and reduction in production costs. As indicated by WP effect size analysis, for > 65%FI, WP either increased significantly or was at par with FI (Fig. 1). The significant gain in WP at all DI levels compared to FI shows that the eggplant is comparatively resilient to water stress. Reviewing the DI effects on vegetables, Singh et al.^3^ also reported better performance of eggplant among the vegetable crops under water deficit conditions. For potato, tomato, and pepper, the highest gain in WP at 35–50%FI suggests that these low DI levels may be advantageous for areas with serious water shortage where crop WP is given more emphasis than yield. However, since vegetables are sold on a fresh weight basis, the quality of produce may be compromised at very low irrigation rates. For instance, Badr et al.^29^ reported a 36% decline in mean tuber weight (ultimately size) for potatoes subjected to 40% ET_c_ irrigation. Similarly, Fabeiro et al.^30^ observed a substantial increase in the percentage of small-sized tubers (< 30 mm diameter) employing 40% ET_c_ based irrigation. In tomatoes, a deficit irrigation has been reported to improve quality attributes, such as total soluble solids, sugars, titrable acidity, ascorbic acid, and color^31,32^. Recently, Darko et al.^33^ observed a significant increase in carbohydrate content in eggplants receiving 70% ET_c_ restoration compared to 100% ET_c_. Such gains are obviously advantageous and may contribute to yield compensation. In tomatoes, significant gain in WP can be achieved with as low as 5.5% reduction in yield using low water deficit irrigation (> 80%FI). Francaviglia and Di Bene^10^ concluded that the decline in yield of processing tomatoes would be marginal at 70–80% of the ET_c_ restoration. A considerable gain in WP (11.3%) with moderate decline in yield (25.6%) under 50–80%FI levels for onion indicates the water-saving potential of these DI levels for onion production in water-limited regions (Fig. 2). Based on the analysis, > 80%FI for cucumbers and > 65%FI for muskmelon seems to be the optimal options to conserve water and sustain the productivity (Fig. 2). Although watermelon recorded the highest gain in WP (47.3%) below 35%FI, the yield penalties were also high (49.7%).

Under low water deficit conditions (> 80%FI), the overall significant differences for yield and WP among the studies and non-significant differences among studies within a crop except tomato, pepper, onion, and watermelon indicate that crop species was the main determinant of yield and WP at this irrigation rate (Supplementary Fig. S1). The interactions of other factors (soil texture, climate, and production system) with irrigation rate became more pronounced towards lower irrigation rates, as indicated by the increasing within crop differences with decreasing irrigation rate. Besides crop species, the major factors that can influence crop yield and WP under DI include irrigation method, genotype, soil amendments, and environmental factors such as, climate and soil characteristics^6^. Evaluation of the interactive effects of these factors and DI can shed light on the differences in yield effects encountered among the studies. In the current meta-analysis, we attempted to quantify the effect of soil texture, climate, and production system (open-field/greenhouse) on yield and WP of vegetable crops subjected to different DI levels.

Across the DI classes, the differences in yield effects among soil textures were significant at < 35%FI and the differences in WP effects were significant at < 35% and 50–65%FI classes (Fig. 3). For all other DI classes, the discrepancies in yield and WP effects due to soil texture were non-significant. Under lower irrigation classes, that is 35–50% and < 35%FI, loam recorded the least decline in yield whereas the gain in WP was highest in clay soils (Fig. 3). This inconsistency in yield and WP trend may be due to the differences in irrigation amounts within the same DI class. Nevertheless, the results suggest that the lower DI rates were more effective in medium to fine textured soils compared to coarse soils. Overall, loam had yield advantage of 12.9 and 7.8% over sand and clay soils, respectively (Supplementary Table S2). Higher water holding capacity seems a probable reason for better yield performance of medium and fine textured soils under severe water deficits. This does not only make more water available in the root zone but also fetches more time for plants to adjust to the water stress. Contrarily, the coarse textured soils having higher infiltration and drainage rates lose water at faster rate leading to water stress shock. Owing to these reasons, fine textured soils utilize rainwater more efficiently compared to coarse soils. The advantage for yield and WP performance of medium to fine compared to coarse textured soils in rainfed or nearly rainfed conditions (< 35%FI) supports the above statement. Now, higher water holding capacity does not necessarily mean greater plant available water^34^. Finer the soil texture, more tightly the soil holds the water. Under low moisture conditions, the plant available water for loam can possibly be greater than clay soil depending on soil moisture content. This explains the yield advantage of loam over clay soil observed in lower DI classes, though the differences were non-significant. These results are in consensus with the findings of potato field experiments conducted by Martin and Miller^19^ and Ahmadi et al.^18^, which showed yield advantage of medium soil textures over coarse soil textures under DI regimes. In a meta-analysis comparing the effects of DI and partial root-zone drying irrigation, Adu et al.^1^ also suggested better performance of fine textured and deep soils. Francaviglia and Di Bene^10^ also observed the yield advantage of fine textured soils over the coarse texture for tomato production under DI. A meta-analysis study on yield and WP of wheat suggested that DI is more advantageous for loamy and sandy soils^15^. A recent meta-analysis study by Cheng et al.^12^ revealed better performance of DI under clay loam though the results were based on a wide range of crops. Our results were in part in consensus with the above-mentioned studies under lower irrigation rates. However, under 50–65%FI, the WP effects were more advantageous for silty clay loam and sand, though comparable to clay, clay loam, loam, loamy sand, and sandy loam. Under 65–80%FI, all soils except sand, loamy sand, and silt loam recorded a significant gain in WP while sandy clay loam was the most advantageous at > 80%FI (Fig. 3). Despite these mixed WP results under moderate to high irrigation rate (> 50%FI), the meta-analysis consistently revealed the yield advantage of medium textured loam over other soil textures under various DI regimes. This meta-analysis assessed the soil texture effects under DI on eight vegetables collectively, but the effect of soil texture on yield under DI may be crop-specific. However, such analysis will be viable only in those crops wherein enough studies are available for various soil textures. Besides texture, the soil electrical conductivity (EC) and pH can also affect the water availability and thus crop yield, but such information was hardly available in the majority of articles included in this systematic review.

In all four climates, the magnitude of yield reductions increased with an increase in water deficit. Near rainfed conditions in < 35%FI level resulted in the highest yield reductions in all climates (Fig. 4). A more severe impact on yield and WP was observed in dry climate at this level as hot and dry conditions multi-folds water stress levels experienced by crop grown with very little supplemental water. At lower water deficit conditions (> 65%FI), yield reductions in dry climate were relatively lower than tropical and subtropical (Fig. 4). Most of the dry climate studies used either drip or sprinkler irrigation systems as compared to flood in tropical and subtropical studies. Flood irrigation system is the least efficient and suffers higher evaporation and runoff loss. Thus, relatively lesser of the applied water was available to crops, which might have a negative effect on crop growth and yield. Also, most of the tropical and subtropical studies were either conducted during a drier season or under a rain shelter. Tropical and subtropical climatic regions receive a relatively higher amount of solar radiation. With low or negligible precipitation, a reduction in supplemental water may have resulted in a higher stressed environment in these climates. At all DI levels, temperate climate with moderate temperature and rainfall had the lowest yield reductions. Across the four climates, increase in WP was substantial under temperate and dry climate. For all other climates, WP under various DI levels remained comparable to FI except the near rainfed condition for the dry climate, wherein WP decreased substantially. A meta-analysis of wheat and maize also reported higher WP under temperate conditions^13^. Overall, the gains in WP under these climates seem to be much lower than those observed in the crop effect analysis. This is partly attributed to the exclusion of greenhouse studies while analyzing climate effects. The open-field conditions showed noticeably smaller gains in WP compared to the greenhouse (Fig. 5). These results suggest that for harsh climatic conditions, the use of DI under protected cultivation of vegetables may be more water productive than open-field. One of the limitations associated with the analysis of climate effect size was that the Koppen-Geiger climate classification maps used in this study might not always represent the true climate during the growing season. For instance, while testing the effects of DI on watermelon under three different environments, Bang et al.^22^ recorded higher overall reference ET during the growing period under temperate climate than semi-arid and subtropical climates. Only a few publications stated the actual climate for the experimental duration, making it difficult to arrive at robust conclusions.

The results indicated yield and WP advantages of DI application to vegetable crop production under greenhouse over open-field at all irrigation levels except the rainfed or nearly rainfed conditions (< 35%FI) (Fig. 5). The use of controlled structures, such as the greenhouse, can reduce the crop water requirement by lowering the ET. The plastic cover lowers the wind speed, reduces solar radiation, entraps longwave radiations, and thus reduce moisture losses^24^. Consequently, the reference ET under the greenhouse is reduced by 65–80% in comparison to open-field conditions^35^. This reduction in water demand under protected cultivation is more pronounced in dry (arid and semi-arid) and tropical climates characterized by low rainfall and high temperature conditions, respectively. Harmanto et al.^36^ found that the ET_c_ for tomatoes inside the greenhouse was 75% of outside in a tropical environment. Under the Mediterranean environment, Stanghellini^37^ observed the actual ET inside the greenhouse to be 70% of that recorded outside. This explains the higher WP gains due to DI under the greenhouse than open-field. Under the least DI level, the greenhouse plants obviously did not receive any rainfall water leading to a drastic reduction in yield and WP. Comparing the impact of different DI levels on cucumber yield between open-field and greenhouse in an arid environment, Alomran, et al.^28^ observed considerably higher yield losses under open-field than greenhouse with the reduction in irrigation rate. The evidence in the literature and the current results suggest that the adaption of protected cultivation in vegetables can save water and sustain productivity, especially under harsh climatic conditions. The integration of rainwater harvesting systems with greenhouse can further help towards the conservation of irrigation water.

### Strengths, limitations, and implications

Our study represents a comprehensive analysis of the effects of DI application on yield and WP of major vegetable crops. The significant heterogeneity among the studies found in the analysis is explored using subgroup analysis of prior defined variables/factors. The estimation of quantitative changes in yield and WP associated with a wide range of irrigation levels for individual vegetable crops, under various soil textures, climatic conditions and production settings provided in this study could assist in decision making while devising limited irrigation strategies in a region. Majority of the studies included in this study provided information on the subgroup factors considered for analysis. However, some confounding factors (e.g. irrigation methods, soil pH, EC, and bulk density) which could be responsible for existing variation were not explored due to the lack of sufficient information available for generating enough pairwise comparisons. Uncertainties exist in such analysis due to numerous other factors, such as field management practices, planting density, nutrient management, irrigation timings/intervals, mulching as well as researcher experiences and preferences. Some of these uncertainties are hard to eliminate. Nevertheless, while extracting data from the studies reporting interaction of irrigation levels with other factors, we considered only the irrigation effects to exclude the variability due to other factors. For instance, if a study presented significant interaction for yield and WP between irrigation levels and mulching, only irrigation effects were included, eliminating the mulching effects.

The sample size of dataset for analyzing the WP effects was smaller than the yield dataset as few studies did not provide sufficient information on rainfall and irrigation. Few subgroups and irrigation levels for some crops could not be included in the analysis due to unavailability of enough pairwise comparisons. Few other subgroup analyses were based on a relatively lesser number of studies reducing the reliability of those results at a broad scale. This meta-analysis determined the feasibility of DI application under different soil textures, climates, and production systems collectively for eight vegetables, but the interactions of these factors with DI may be crop-specific. However, such analysis will be viable only in those crops wherein enough studies are available for generating a reasonable number of pairwise comparisons. As a number of new studies investigating limited irrigation responses of vegetables are being added to the literature every year, an in-depth analysis focusing on an individual vegetable crop can potentially be performed in the future.

## Conclusions

In general, greater than moderate level of water deficits (< 65%FI) have adverse effects on vegetable yield regardless of external factors, such as soil texture, climate, and production system. Although the decline in yield was statistically significant even under low water deficit conditions, these irrigation levels are justifiable for regions already undergoing or foreseeing water restrictions. In light of declining water resources around the globe, water restrictions are inevitable. In such a scenario, increased WP, decreased cost of production and enhanced quality of produce under DI may contribute towards yield compensation. Considering the yield losses and gains in WP, irrigation rates may be assigned depending on water availability. Based on the WP gains, irrigation levels as low as 35–50%FI for potatoes, tomato, and pepper, and 50–65% for onion and even < 35%FI for eggplant and watermelon seem to be an option for areas with severe water scarcity. However, higher water deficits can also have an adverse effect on quality in terms of reduction in fruit/tuber size, as suggested by some previous studies. For many vegetables, information on qualitative aspects as affected DI levels is still lacking.

The soil texture effects on vegetable yield were significant only under near-rainfed (< 35%FI) wherein medium to fine textured soils were advantageous over coarse soils. Overall, loam soil recorded the least reduction in yield under almost all DI levels. In open-field production system, deficit irrigation was most beneficial under temperate climate especially at moderate DI (50–80%FI). The DI application under greenhouse recorded lesser yield reductions compared to open-field conditions. The change in WP followed a similar pattern under both greenhouse and open-field, but magnitude of WP gains was considerably greater for greenhouse conditions. These results, along with climatic effects, suggest that the adaption of protected vegetable production is advantageous for water conservation, especially for harsh climatic conditions characterized by low rainfall and high temperature.

A majority of studies included in this meta-analysis did not provide standard deviation (SD) or standard error (SE) values. The SDs for these studies were interpolated using SD values from other studies within each crop. We encourage the researchers to include these measures of dispersion of the data. We also encourage the inclusion of some other important details related to the experiments, such as soil characteristics (texture, pH, EC, organic matter), climatic conditions, and irrigation methods.

## Methods

This study followed Cochrane recommendations^38^ and Preferred Reporting Items for a Systematic Review and Meta-analysis (PRISMA) guidelines^39^. The PRISMA checklists are given in the supplementary information (Table S4 and S5).

### Data collection

We searched peer-reviewed literature for studies that reported yield differences in vegetable crops subjected to deficit irrigation or moisture stress. The search was conducted on Google Scholar and Web of Science using the different combinations of the following keywords: vegetable yield and deficit irrigation or moisture stress or water stress or drought stress. Based on the availability of literature, eight major vegetable crops, potato (*Solanum tuberosum* L.), tomato (*Solanum lycopersicum*) L.), pepper (*Capsicum annuum* L.), eggplant (*Solanum melongena* L.), onion (*Allium cepa* L.), cucumber (*Cucumis sativus* L.), muskmelon (*Cucumis melo* L.) and watermelon (*Citrullus lanatus* L.) were identified for inclusion in the meta-analysis. Thereafter, a thorough search was conducted for individual crops. We adopted the following criteria for inclusion of studies: should be an open-field or greenhouse plot study (pot studies were excluded), provided yield data for FI and DIs; provided detailed information on irrigation criteria and DI was given as a percentage of FI; provided information on soil texture of experimental site or percentage of sand, silt and clay particles in the soil; provided mean yield and sample size for individual irrigation levels. In majority of these studies, DIs were based on ET_c_ or pan evaporation with few studies using soil water content as irrigation criteria.

The WP was obtained directly (if provided) or calculated as:

1 $$\mathrm{WP}=\mathrm{Yield}/(\mathrm{Irrigation}+\mathrm{Rainfall})$$

Few studies did not provide rainfall and irrigation information rendering the number of observations for WP data lesser than yield data.

The sample size (N), mean and SD are the three major input variables for estimating effect size in a meta-analysis^40^. The sample size was the same as the number of replications in most cases with few exceptions, where multiple factors were involved. The mean yield/WP and SE or SD were obtained from tables or extracted from graphs provided in the publications using GetData Graph Digitizer 2.26. If SD values were not provided directly, it was calculated from SE using Eq. (2) as described by Higgins et al.^41^.

2 $$\mathrm{SD}=\mathrm{SE }\times \surd \mathrm{N}$$

If the SD was not given and could not be computed from the given information, it was estimated using the coefficient of variation (CV) of studies within the same crop calculated using Eq. (3) as described by Wiebe et al.^42^.

3 $$\mathrm{CV}=\mathrm{SD}/\mathrm{Mean }\times 100$$

The CV_mean_ was calculated as the mean of CVs of studies obtained using Eq. (3). The missing SD (SD_missing_) was then calculated using the CV_mean_ as in Eq. (4).

4 $$\mathrm{WP}=(\mathrm{CV}_{\text{mean}} \times \mathrm{Mean}_{\text{missingSD}})/100$$

where Mean_missing SD_ is the mean of the corresponding observations with missing SD.

Soil texture was determined from the sand, silt, and clay percentage (if provided) using the United States Department of Agriculture-Natural Resources Conservation Service Soils soil texture calculator^43^ or obtained directly from the publication. Most of the studies provided this information with a few exceptions that were excluded while conducting the meta-analysis for soil textures. While comparing the DI effects under open-field and greenhouse conditions, only the crops having at least one greenhouse plot study were included. Thus, potato and watermelon crops were excluded for not having any greenhouse plot study. For assessing the yield effects under different climatic zones, only open-field studies were included. Based on the location (latitudes and longitudes), the studies were categorized into four climate zones: dry, temperate, subtropical, and tropical. Updated Koppen-Geiger climate classification maps of Beck et al.^44^ were used to arrive at the climate of a study.

For studies, where authors reported N, mean, and SDs separately for different years/seasons or subgroup treatments, the data were combined into a single group for each irrigation treatment. For instance, if a single study presented the yield data separately for multi-seasons or multi-years, we combined the years by adding sample sizes and calculating the mean yield. The combined SD (SD_combined_) was obtained by using the Eq. (5):

5 $${\text{SD}}_{\text{combined}}\,{=}\,\sqrt{\frac{\left({\text{N}}_{1}\text{-1}\right){\text{S}}{\text{D}}_{1}^{2}\text{+}\left({\text{N}}_{2}\text{-1}\right){\text{S}}{\text{D}}_{2}^{2}\text{+}\frac{{\text{N}}_{1}{{\text{N}}}_{2}}{{\text{N}}_{1}\text{+}{\text{N}}_{2}}\left({\text{M}}_{1}^{2}\text{+}{\text{M}}_{2}^{2}\text{-2}{\text{M}}_{1}{{\text{M}}}_{2}\right)}{{\text{N}}_{1}\text{+}{\text{N}}_{2}\text{-1}}}$$

where N_1_ and N_2_ are number of observations (sample size) in group 1 and group 2, respectively; M_1_ and M_2_ are means, and SD_1_ and SD_2_ are standard deviations of group 1 and group 2, respectively.

However, for multi-location studies representing different soil textures or climatic conditions, yield results from each location were examined independently in the meta-analysis. Also, for instances, where the same DI class included two or more irrigation levels, the levels were included as independent cases.

### Data arrangement and effect size analysis

Prior to analysis, we arranged the data separately for following DI level classes given as percentage of FI (100%): < 35%; 35–50%; 50–65%; 65–80% and > 80%. The meta-analysis was conducted distinctly for comparison of the yield and WP effect size of each DI class against FI.

Effect size, a type of standardized mean difference, is a general statistical parameter that enables the comparison of results from different studies on the same scale^41^. Different measures for effect size include Cohen’s d, Hedges’ g, and Glass’s delta. In this study, we used Hedges’ g^40^ for assessing the magnitude of the effect of DI levels on vegetable crop yield. Hedges’ g uses pooled SD (SD_pooled_) and computes the effect size of paired groups as:

6 $${\text{g}} = \left( {\text{M}_\text{e}} - {\text{M}_\text{c}} \right)/{\text{SD}}_{\text{pooled}}$$

where M_e_ and M_c_ are the means of the active group (DI level) and control group (FI), respectively.

The MetaXL version 5.3^45^ was employed to conduct the statistical analysis using the random effect model at a 95% confidence interval (CI). The possibility of any publication bias in the yield and WP data was examined using funnel plots^46^ and sensitivity analyses were performed to assess the influence of individual observations on mean effect sizes and corrected for biases. Several distinct meta-analyses were conducted to assess the influence of different variables including, crop species, soil texture, production system (open-field or greenhouse), and climate on yield and WP effects under different DI levels. Any irrigation level or considered variables having less than two comparisons were excluded from the analysis. For each meta-analysis, FI (100%) was defined as control. A positive Hedges’ g value indicated an increase in yield/WP for DI over FI and a negative value indicated a reduction in yield/WP under DI compared to FI. The significance of effect size is determined by the output CI. An overlapping CI indicated no significant difference whereas non-overlapping CI indicated a significant difference (p < 0.05) among studies or subgroups. Figures 1, 2, 3, 4 and 5 were created using SigmaPlot Version 14.0 (Systat Software, San Jose, CA). The supplementary figures S1-S10 were created using MetaXL version 5.3 and supplementary figure S11 was created with ArcMAP version 10.8 (Environmental Systems Research Institute Inc., Redlands, CA).

To quantify yield and WP response of a treatment vs. control (FI), percentage change in the means between the two was calculated. This was obtained by using Eq. (7):

7 $$\mathrm{\% change}=\frac{\sum \frac{{\mathrm{w}}_{\mathrm{n}}\left({\mathrm{Y}}_{\mathrm{n}}-{\mathrm{y}}_{\mathrm{n}}\right)\times 100}{{\mathrm{Y}}_{\mathrm{n}}}}{100}$$

where w_n_, y_n_, and Y_n_, represents weightage factor obtained from the effect size analysis, treatment mean, and control (FI) mean of the n^th^ study, respectively. The percentage change in yield and WP under various deficit irrigation levels compared to FI as affected by crops species, soil texture, climate, and production system is given in supplementary table S1 and S2.

## Supplementary Information

Supplementary Information.

## Data Availability

Most of the data analyzed in this study are included in the article and its supplementary information files. The datasets not included in the published article are available from the corresponding author on reasonable request.
