# Supplementary material for: A global meta-analysis of yield and water productivity responses of vegetables to deficit irrigation
Source: Sci Rep. 2021 Nov 11;11:22095. doi: 10.1038/s41598-021-01433-w (PMC8585919; doi:10.1038/s41598-021-01433-w)
Supplement: Supplementary file 1 — Supplementary Information. [file 41598_2021_1433_MOESM1_ESM.docx]

**A global meta-analysis of yield and water productivity responses of vegetables to deficit irrigation**

Manpreet Singh^a^, Paramveer Singh^b^, Sukhbir Singh*^a^, Rupinder Kaur Saini^a^ and Sangamesh V. Angadi^b^

*^a^Department of Plant and Soil Science, Texas Tech University, Lubbock, TX 79409, USA.*

*^b^Department of Plant and Environmental Sciences, New Mexico State University, Las Cruces, NM 88003, USA.*

**Corresponding author*

*Sukhbir Singh:* [*s.singh@ttu.edu*](mailto:s.singh@ttu.edu)

Table S1: Overview of studies included in the meta-analysis

| **Reference** | **Crop** | **Country** | **Soil** | **Climate** | **Experiment location** |
| --- | --- | --- | --- | --- | --- |
| Şimşek et al. (2004) ^1^ | Cucumber | Turkey | Clay | Temperate | Open-field |
| Kirnak and Demirtas (2006) ^2^ | Cucumber | Turkey | Clay loam | Temperate | Open-field |
| Ertek et al. (2006) ^3^ | Cucumber | Turkey | Loam | Temperate | Open-field |
| Ayas and Demirtas (2009) ^4^ | Cucumber | Turkey | Sandy loam | Temperate | Greenhouse |
| Wang et al. (2009) ^5^ | Cucumber | China | Loam | Temperate | Greenhouse |
| Amer et al. (2009) ^6^ | Cucumber | Egypt | Clay loam | Dry | Open-field |
| Zhang et al. (2011) ^7^ | Cucumber | China | Loam | Temperate | Greenhouse |
| Alomran et al. (2013) ^8^ | Cucumber | Saudi Arabia | Loamy sand | Subtropical | Greenhouse |
| Rahil and Qanadillo (2015) ^9^ | Cucumber | Palestine | Clay | Subtropical | Greenhouse |
| Sahin et al. (2015) ^10^ | Cucumber | Turkey | Clay loam | Temperate | Open-field |
| Çakir et al. (2017) ^11^ | Cucumber | Turkey | Silty loam | Subtropical | Greenhouse |
| Abd El-Mageed et al. (2018) ^12^ | Cucumber | Egypt | Loamy sand | Subtropical | Open-field |
| Wang et al. (2019) ^13^ | Cucumber | China | Silt loam | Temperate | Greenhouse |
| Wang et al. (2019) ^14^ | Cucumber | China | NA | Temperate | Greenhouse |
| Abdelraouf et al. (2020) ^15^ | Cucumber | Egypt | Sand | Dry | Greenhouse |
| Ali et al. (2020) ^16^ | Cucumber | China | NA | Temperate | Greenhouse |
| Zakka et al. (2020) ^17^ | Cucumber | Nigeria | NA | Temperate | Open-field |
| Ghahremani et al. (2020) ^18^ | Cucumber | Iran | Clay loam | Dry | Open-field |
| He et al. (2021) ^19^ | Cucumber | China | NA | Temperate | Greenhouse |
| Parkash et al. (2021) ^20^ | Cucumber | US | Sandy clay loam | Dry | Open-field |
|  |  |  |  |  |  |
| Chartzoulakis and Drosos (1995) ^21^ | Eggplant | Crete | Sandy loam | Temperate | Greenhouse |
| Kirnak et al. (2002) ^22^ | Eggplant | Turkey | Clay loam | Temperate | Open-field |
| Senyigit et al. (2011) ^23^ | Eggplant | Turkey | Sandy loam | Temperate | Greenhouse |
| Demirel et al. (2014) ^24^ | Eggplant | Turkey | Clay | Temperate | Open-field |
| Díaz-Pérez and Eaton (2015) ^25^ | Eggplant | US | Sandy loam | Subtropical | Open-field |
| Karajeh and Mohawesh (2016) ^26^-1 | Eggplant | Jordan | Silty loam | Dry | Open-field |
| Karajeh and Mohawesh (2016) ^26^-2 | Eggplant | Jordan | Clay loam | Dry | Open-field |
| Karam and Nangia (2016) ^27^ | Eggplant | Lebanon | Clay | Temperate | Open-field |
| Mohawesh (2016) ^28^-1 | Eggplant | Jordan | Sandy loam | Subtropical | Open-field |
| Mohawesh (2016) ^28^-2 | Eggplant | Jordan | Clay loam | Dry | Open-field |
| Çolak et al. (2017) ^29^ | Eggplant | Turkey | Clay loam | Temperate | Open-field |
| Abdrabbo et al. (2017) ^30^ | Eggplant | Egypt | Clay | Dry | Greenhouse |
| Ayas (2017) ^31^ | Eggplant | Turkey | Sandy loam | Temperate | Greenhouse |
| Darko et al. (2019) ^32^ | Eggplant | Ghana | Sandy clay loam | Tropical | Open-field |
| Bader et al. (2020) ^33^ | Eggplant | Iraq | Sandy loam | Dry | Greenhouse |
| Badr et al. (2020) ^34^ | Eggplant | Egypt | Loamy sand | Dry | Open-field |
| Ibrahim et al. (2020) ^35^ | Eggplant | Sudan | Sandy clay loam | Dry | Open-field |
| Abd El-Mageed et al. (2021) ^36^ | Eggplant | Egypt | Sandy loam | Dry | Open-field |
| Ebrahimi et al. (2021) ^37^ | Eggplant | Iran | Loam | Dry | Open-field |
| Mwinuka et al. (2021) ^38^ | Eggplant | Tanzania | Sand clay loam | tropical | Open-field |
| Rodan et al. (2021) ^39^ | Eggplant | Iran | Sandy loam | Dry | Open-field |
| Semida et al. (2021) ^40^ | Eggplant | Egypt | Sandy loam | Dry | Open-field |
|  |  |  |  |  |  |
| Fabeiro et al. (2002) ^41^ | Muskmelon | Spain | Sandy clay loam | Temperate | Open-field |
| Kirnak et al. (2005) ^42^ | Muskmelon | Turkey | Clay loam | Temperate | Open-field |
| Dogan et al. (2007) ^43^ | Muskmelon | Turkey | Clay | Temperate | Open-field |
| Sensoy et al. (2007) ^44^ | Muskmelon | Turkey | Loamy sand | Continental | Open-field |
| Cabello et al. (2009) ^45^ | Muskmelon | Spain | Sandy loam | Temperate | Open-field |
| Zeng et al. (2009) ^46^ | Muskmelon | China | NA | Subtropical | Greenhouse |
| Al-Mefleh et al. (2012) ^47^ | Muskmelon | Jordan | Clay loam | Temperate | Open-field |
| Ahmadi-Mirabad et al. (2014) ^48^ | Muskmelon | Iran | Loam | Temperate | Open-field |
| Alenazi et al. (2015) ^49^ | Muskmelon | Saudi Arabia | Sand | Dry | Greenhouse |
| Sharma et al. (2016) ^50^ | Muskmelon | US | Silty clay | Subtropical | Open-field |
| Barzegar et al. (2018) ^51^ | Muskmelon | Iran | Clay loam | Dry | Open-field |
| Akhoundnejad and Dasgan (2020) ^52^ | Muskmelon | Turkey | NA | Temperate | Open-field |
| Ezzo et al. (2020) ^53^ | Muskmelon | Egypt | Clay | Dry | Green house |
| Yavuz et al. (2021) ^54^ | Muskmelon | Turkey | Silty clay loam | Dry | Open-field |
| Yavuz (2021) ^55^ | Muskmelon | Turkey | Silty clay loam | Dry | Open-field |
| Zeineldin and Al-Molhim (2021) ^56^ | Muskmelon | Saudi Arabia | Sand | Dry | Open-field |
|  |  |  |  |  |  |
| Bekele and Tilahun (2007) ^57^ | Onion | Ethiopia | NA | Subtropical | Open-field |
| Kumar et al. (2007) ^58^ | Onion | India | Sandy loam | Dry | Open-field |
| Patel and Rajput (2008) ^59^ | Onion | India | Sandy loam | Dry | Open-field |
| Ayas and Demirtaș (2009) ^60^ | Onion | Turkey | Sandy loam | Temperate | Greenhouse |
| Enciso et al. (2009) ^61^ | Onion | US | Sandy clay loam | Temperate | Open-field |
| Nagaz et al. (2012) ^62^ | Onion | Tunisia | Sand | Dry | Open-field |
| Igbadun et al. (2012) ^63^ | Onion | Nigeria | Loam | tropical | Open-field |
| Leskovar et al. (2012) ^64^ | Onion | US | Silty clay | Subtropical | Open-field |
| Zheng et al. (2012) ^65^ | Onion | China | Sandy loam | Dry | Open-field |
| Patel and Rajput (2013) ^66^ | Onion | India | Sandy loam | Dry | Open-field |
| Rop et al. (2016) ^67^ | Onion | Kenya | Sandy loam | Tropical | Open-field |
| Enchalew et al. (2016) ^68^ | Onion | Ethiopia | Clay loam | tropical | Open-field |
| Wakchaure et al. (2018) ^69^ | Onion | India | Sandy clay | Dry | Open-field |
| Afzal et al. (2020) ^70^ | Onion | Pakistan | Sandy loam | Dry | Open-field |
| Ambomsa and Seyoum (2019) ^71^ | Onion | Ethiopia | Loam | Temperate | Open-field |
| Dingre and Pawar (2020) ^72^ | Onion | India | Sandy clay loam | Dry | Open-field |
| Hefzy et al. (2020) ^73^ | Onion | Egypt | Sand | Dry | Open-field |
| Kandongo et al. (2020) ^74^ | Onion | South Africa | NA | Dry | Open-field |
| Mugoro et al. (2020) ^75^ | Onion | Ethiopia | Clay loam | Dry | Open-field |
| Nurga et al. (2020) ^76^ | Onion | Ethiopia | Clay loam | Dry | Open-field |
| Piri and Naserin (2020) ^77^ | Onion | Iran | Sandy loam | Dry | Open-field |
| Semida et al. (2020) ^78^ | Onion | Egypt | Sandy loam | Dry | Open-field |
| Shirzadi et al. (2020) ^79^ | Onion | Iran | Sandy loam | Dry | Open-field |
| El–Metwally et al. (2021) ^80^ | Onion | Egypt | Sand | Dry | Open-field |
| Tegenu (2021) ^81^ | Onion | Ethiopia | Clay | Temperate | Open-field |
| Wakchaure et al. (2021) ^82^ | Onion | India | Sandy clay | Dry | Open-field |
|  |  |  |  |  |  |
| Chartzoulakis and Drosos (1997) ^83^ | Pepper | Crete | Sandy loam | Temperate | Greenhouse |
| González-Dugo et al. (2007) ^84^ | Pepper | Spain | Silty clay | Temperate | Open-field |
| Guang-Cheng et al. (2008) ^85^ | Pepper | China | Clay loam | Subtropical | Greenhouse |
| Gadissa and Chemeda (2009) ^86^ | Pepper | Ethiopia | Sandy clay loam | Subtropical | Open-field |
| Karam et al. (2009) ^87^ | Pepper | Lebanon | Clay | Temperate | Open-field |
| Guang-Cheng et al. (2010) ^88^ | Pepper | China | Clay loam | Subtropical | Greenhouse |
| AlHarbi et al. (2013) ^89^ | Pepper | Saudi Arabia | Sand | Dry | Greenhouse |
| Demirel et al. (2014) ^24^ | Pepper | Turkey | Clay loam | Temperate | Open-field |
| Ćosić et al. (2015) ^90^ | Pepper | Serbia | Clay loam | Subtropical | Open-field |
| Kuşçu et al. (2016) ^91^ | Pepper | Turkey | Clay loam | Temperate | Open-field |
| Celebi (2018) ^92^ | Pepper | Turkey | Clay | Dry | Open-field |
| Sezen et al. (2019) ^93^ | Pepper | Turkey | Silty clay | Temperate | Open-field |
| Abdelkhalik et al. (2019) ^94^ | Pepper | Spain | Silt loam | Subtropical | Open-field |
| Badawi et al. (2020) ^95^ | Pepper | Egypt | Sandy loam | Dry | Open-field |
| Demir and Özbahçe (2021) ^96^ | Pepper | Turkey | Clay | Temperate | Open-field |
| Gisbert-Mullor et al. (2020) ^97^ | Pepper | Spain | Sandy loam | Mediterranean | Greenhouse |
| Sumathi et al. (2020) ^98^ | Pepper | India | Silt loam | Dry | Greenhouse |
| Kabir et al. (2021) ^99^ | Pepper | US | Sandy loam | Subtropical | Open-field |
|  |  |  |  |  |  |
| Martin and Miller (1983) ^100^-1 | Potato | US | Loam | Dry | Open-field |
| Martin and Miller (1983) ^100^-2 | Potato | US | Sand | Dry | Open-field |
| Shock et al. (1998) ^101^ | Potato | US | Silt loam | Continental | Open-field |
| Fabeiro et al. (2001) ^102^ | Potato | Spain | Sandy clay loam | Temperate | Open-field |
| Alva et al. (2002) ^103^ | Potato | US | Sand | Dry | Open-field |
| Yuan et al. (2003) ^104^ | Potato | Japan | Silt loam | Subtropical | Open-field |
| Onder et al. (2005) ^105^-1 | Potato | Turkey | Clay loam | Temperate | Open-field |
| Onder et al. (2005) ^105^-2 | Potato | Turkey | Loam | Temperate | Open-field |
| Kiziloglu et al. (2006) ^106^ | Potato | Turkey | Loam | Continental | Open-field |
| Ierna and Mauromicale (2006) ^107^ | Potato | Italy | Clay loam | Temperate | Open-field |
| Shahnazari et al. (2008) ^108^ | Potato | Denmark | Sand | Temperate | Open-field |
| Alva et al. (2008) ^109^ | Potato | US | Sand | Dry | Open-field |
| Ahmadi et al. (2010) ^110^-1 | Potato | Denmark | Sandy loam | Temperate | Open-field |
| Ahmadi et al. (2010) ^110^-2 | Potato | Denmark | Loamy sand | Temperate | Open-field |
| Ahmadi et al. (2010) ^110^-3 | Potato | Denmark | Sand | Temperate | Open-field |
| Badr et al. (2010) ^111^ | Potato | Egypt | Loamy sand | Dry | Open-field |
| Ayas and Korukçu (2010) ^112^ | Potato | Turkey | Sandy clay loam | Temperate | Open-field |
| Badr et al. (2012) ^113^ | Potato | Egypt | Loamy sand | Dry | Open-field |
| Ierna and Mauromicale (2012) ^114^ | Potato | Italy | Clay loam | Temperate | Open-field |
| Ahmadi et al. (2014) ^115^ | Potato | Iran | Clay loam | Dry | Open-field |
| Maralian et al. (2014) ^116^ | Potato | Iran | Clay loam | Continental | Open-field |
| Ghazouani et al. (2015) ^117^ | Potato | Tunisia | Sandy loam | Dry | Open-field |
| Mokh et al. (2015) ^118^ | Potato | Tunisia | Sand | Dry | Open-field |
| Nouri et al. (2016) ^119^ | Potato | Iran | NA | Dry | Open-field |
| Ghazouani et al. (2017) ^120^ | Potato | Tunisia | Sandy loam | Temperate | Open-field |
| Zin El-Abedin et al. (2017) ^121^ | Potato | Saudi Arabia | Sandy loam | Dry | Open-field |
| Barakat et al. (2020) ^122^ | Potato | Egypt | Clay loam | Dry | Open-field |
| El Youssfi et al. (2020) ^123^ | Potato | Morocco | Silty clay loam | Temperate | Open-field |
| Elmetwalli and Elnemr (2020) ^124^ | Potato | Egypt | Sandy loam | Dry | Open-field |
| Abd El-Wahed et al. (2020) ^125^ | Potato | Egypt | Sandy loam | Dry | Open-field |
| Essah et al. (2020) ^126^ | Potato | US | Sandy loam | Dry | Open-field |
| Gogoi et al. (2020) ^127^ | Potato | India | Sandy clay loam | Subtropical | Open-field |
| Kassaye et al. (2020) ^128^ | Potato | Ethiopia | Clay loam | Dry | Open-field |
| Mattar et al. (2021) ^129^ | Potato | Saudi Arabia | Sandy loam | arid | Open-field |
| O’Shaughnessy et al. (2020) ^130^ | Potato | Texas | Clay loam | Dry | Open-field |
| Zahran et al. (2020) ^131^ | Potato | Egypt | Sand | Dry | Open-field |
| Al-Shamary et al. (2021) ^132^ | Potato | Iraq | Sandy clay loam | Dry | Open-field |
|  |  |  |  |  |  |
| Obreza et al. (1996) ^133^ | Tomato | US | Sand | Subtropical | Open-field |
| Kirda et al. (2004) ^134^ | Tomato | Turkey | Clay | Temperate | Greenhouse |
| Harmanto et al. (2005) ^135^ | Tomato | Thailand | Clay loam | Tropical | Greenhouse |
| del Amor and del Amor (2007) ^136^ | Tomato | Brazil | Clay loam | Tropical | Open-field |
| Singh et al. (2009) ^137^ | Tomato | India | Sandy loam | Dry | Open-field |
| Patanè and Cosentino (2010) ^138^-1 | Tomato | Italy | Sandy loam | Temperate | Open-field |
| Patanè and Cosentino (2010) ^138^-2 | Tomato | Italy | Loam | Temperate | Open-field |
| Ozbahce and Tari (2010) ^139^ | Tomato | Turkey | Clay | Dry | Open-field |
| Wahb-Allah et al. (2011) ^140^ | Tomato | Saudi Arabia | Loamy sand | Dry | Greenhouse |
| Patanè et al. (2011) ^141^ | Tomato | Italy | Sandy loam | Temperate | Open-field |
| Mahadeen et al. (2011) ^142^ | Tomato | Jordan | Sandy clay loam | Subtropical | Open-field |
| Hassan and Abuarab (2013) ^143^ | Tomato | Egypt | Sandy clay loam | Dry | Open-field |
| Kuşçu et al. (2014) ^144^ | Tomato | Turkey | Clay loam | Subtropical | Open-field |
| Wahb-Allah et al. (2014) ^145^ | Tomato | Saudi Arabia | Sand | Dry | Greenhouse |
| Ibrahim et al. (2014) ^146^ | Tomato | Saudi Arabia | sand | Dry | Greenhouse |
| Biswas et al. (2015) ^147^ | Tomato | Bangladesh | Silt clay loam | tropical | Open-field |
| Kumar et al. (2015) ^148^ | Tomato | India | NA | Dry | Open-field |
| Wang et al. (2015) ^149^ | Tomato | China | Silt loam | Temperate | Greenhouse |
| Lahoz et al. (2016) ^150^ | Tomato | Spain | NA | Temperate | Open-field |
| Nangare et al. (2016) ^151^ | Tomato | India | NA | Dry | Open-field |
| Cantore et al. (2016) ^152^ | Tomato | Italy | Sandy clay loam | Temperate | Open-field |
| Bowles et al. (2016) ^153^ | Tomato | US | Loam | Dry | Open-field |
| Djurović et al. (2016) ^154^ | Tomato | Serbia | Clay loam | Temperate | Open-field |
| Xiukang and Yingying (2016) ^155^ | Tomato | China | NA | Dry | Greenhouse |
| Du et al. (2017) ^156^ | Tomato | China | NA | Temperate | Greenhouse |
| Wang and Xing (2017) ^157^ | Tomato | China | Loam | Temperate | Greenhouse |
| Agbna et al. (2017) ^158^ | Tomato | China | Loam | Subtropical | Greenhouse |
| Giuliani et al. (2017) ^159^ | Tomato | Italy | Loam | Temperate | Open-field |
| Abdelhady et al. (2017) ^160^ | Tomato | Egypt | Clay | Dry | Open-field |
| Zhang et al. (2017) ^161^ | Tomato | China | Loam | Dry | Open-field |
| Hashem et al. (2018) ^162^ | Tomato | Saudi Arabia | Sandy loam | Dry | Open-field |
| Al-Shmmari et al. (2020) ^163^ | Tomato | Iraq | Silty loam | Dry | Open-field |
| Abd El- Aziz (2020) ^164^ | Tomato | Egypt | Loamy sand | Dry | Open-field |
| Kumsa (2020) ^165^ | Tomato | Ethiopia | Sandy clay loam | Temperate | Open-field |
| Mattar et al. (2019) ^166^ | Tomato | Saudi Arabia | Sandy loam | Dry | Open-field |
| Mendonça et al. (2020) ^167^ | Tomato | Brazil | Clay | Tropical | Greenhouse |
| Milkereit et al. (2019) ^168^ | Tomato | US | Silty clay loam | Dry | Open-field |
| Patanè et al. (2020) ^169^ | Tomato | Italy | Clay loam | Temperate | Open-field |
| Shabbir et al. (2020) ^170^ | Tomato | China | Clay | Temperate | Greenhouse |
| Zahid et al. (2020) ^171^ | Tomato | Pakistan | Sandy loam | Dry | Greenhouse |
| Wu et al. (2021) ^172^ | Tomato | China | Clay loam | Dry | Greenhouse |
|  |  |  |  |  |  |
| Erdem et al. (2001) ^173^ | Watermelon | Turkey | Clay | Subtropical | Open-field |
| Leskovar et al. (2002) ^174^ | Watermelon | US | NA | Subtropical | Open-field |
| Bang et al. (2004) ^175^-1 | Watermelon | US | Silty clay loam | Subtropical | Open-field |
| Bang et al. (2004) ^175^-2 | Watermelon | US | Sandy loam | Temperate | Open-field |
| Bang et al. (2004) ^175^-3 | Watermelon | US | Loam | Dry | Open-field |
| Şimşek et al. (2004) ^1^ | Watermelon | Turkey | Clay | Dry | Open-field |
| Kirnak et al. (2009) ^176^ | Watermelon | Turkey | Clay | Temperate | Open-field |
| Özmen et al. (2015) ^177^ | Watermelon | Turkey | Clay | Temperate | Open-field |
| Kuşçu et al. (2017) ^178^ | Watermelon | Turkey | Clay loam | Temperate | Open-field |
| Yoosefzadeh Najafabadi et al. (2018) ^179^ | Watermelon | China | Clay loam | Dry | Open-field |
| Abdelkhalik et al. (2019) ^180^ | Watermelon | Spain | Silt loam | Subtropical | Open-field |
| Pawar et al. (2019) ^181^ | Watermelon | India | Clay | Dry | Open-field |
| Enyew et al. (2020) ^182^ | Watermelon | Ethiopia | Clay | Temperate | Open-field |
| khalifa (2020) ^183^ | Watermelon | Egypt | Sand | Dry | Open-field |
| Qin and Leskovar (2020) ^184^ | Watermelon | US | Clay | Temperate | Open-field |
| Yavuz et al. (2020) ^185^ | Watermelon | Turkey | Silty clay loam | Dry | Open-field |

NA data not available

| Table S2: Percentage decline in yield under various deficit irrigation levels compared to full irrigation (FI) as affected by crop species, soil texture, climate, and production system | | | | | |
| --- | --- | --- | --- | --- | --- |
|  | **>80%FI** | **65-80%FI** | **50-65%FI** | **35-50%FI** | **<35%FI** |
| **Crops** |  |  |  |  |  |
| **Cucumber** | 5.0 | 9.6 | 25.1 | 43.5 | NA |
| **Muskmelon** | 4.7 | 9.6 | 28.7 | 37.5 | 55.5 |
| **Eggplant** | 6.8 | 11.5 | 31.8 | 42.4 | 36.5 |
| **Onion** | 8.2 | 13.0 | 25.6 | 36.9 | 77.1 |
| **Pepper** | 14.6 | 17.5 | 29.2 | 35.0 | 35.5 |
| **Potato** | 8.9 | 7.1 | 24.8 | 32.1 | 35.4 |
| **Tomato** | 5.5 | 10.3 | 24.2 | 32.2 | 67.7 |
| **Watermelon** | 1.4 | 3.9 | 17.8 | 53.9 | 49.7 |
| **Soil texture** |  |  |  |  |  |
| **Sand** | 5.3 | 19.2 | 26.1 | 39.1 | 63.1 |
| **Loamy sand** | 11.4 | 21.4 | 27.3 | 42.8 | NA |
| **Sandy loam** | 18.8 | 10.9 | 29.8 | 43.0 | 48.9 |
| **Sandy clay loam** | 8.6 | 11.1 | 34.3 | 39.8 | NA |
| **Sandy clay** | 7.1 | NA | 29.7 | NA | 82.1 |
| **Loam** | 3.9 | 6.2 | 23.0 | 28.5 | 26.9 |
| **Silt loam** | NA | 10.8 | 25.4 | NA | 38.2 |
| **Clay loam** | 9.1 | 6.3 | 24.5 | 47.6 | 44.1 |
| **Silty clay loam** | NA | 15.0 | 30.4 | NA | 71.7 |
| **Silty clay** | NA | 8.7 | 25.9 | NA | NA |
| **Clay** | 9.4 | 9.2 | 22.57 | 37.6 | 48.7 |
| **Climate** |  |  |  |  |  |
| **Dry** | 6.2 | 12.2 | 27.3 | 39.4 | 64.2 |
| **Subtropical** | 12.0 | 17.5 | 30.3 | NA | 41.9 |
| **Temperate** | 10.1 | 6.9 | 17.5 | 36.9 | 38.3 |
| **Tropical** | 7.8 | 16.1 | 33.9 | NA | NA |
| **Production system** |  |  |  |  |  |
| **Greenhouse** | 6.3 | 8.8 | 25.4 | 35.5 | 77.7 |
| **Open**-**field** | 7.8 | 13.3 | 25.8 | 38.3 | 57.2 |

NA data not available

| Table S3: Percentage change in water productivity (WP) under various deficit irrigation levels compared to full irrigation (FI) as affected by crop species, soil texture, climate, and production system | | | | | |
| --- | --- | --- | --- | --- | --- |
|  | **>80%FI** | **65-80%FI** | **50-65%FI** | **35-50%FI** | **<35%FI** |
| **Crops** |  |  |  |  |  |
| **Cucumber** | 15.8 | 13.8 | 8.3 | 25.2 | NA |
| **Muskmelon** | 5.7 | 17.3 | 14.7 | 26.9 | 20.3 |
| **Eggplant** | 12.6 | 14.6 | 23.2 | 29.1 | 49.3 |
| **Onion** | 5.8 | 7.7 | 11.3 | 29.1 | -16.0 |
| **Pepper** | 0.9 | 6.5 | 5.2 | 44.2 | 20.5 |
| **Potato** | 6.2 | 16.6 | 13.5 | 23.2 | 11.2 |
| **Tomato** | 9.8 | 18.7 | 25.6 | 33.3 | 18.3 |
| **Watermelon** | NA | 22.6 | 31.2 | NA | 47.3 |
| **Soil texture** |  |  |  |  |  |
| **Sand** | 11.0 | 10.6 | 28.9 | 32.3 | NA |
| **Loamy sand** | 8.6 | 8.0 | 22.5 | 32.5 | NA |
| **Sandy loam** | 8.6 | 13.9 | 13.2 | 30.4 | 8.2 |
| **Sandy clay loam** | 13.6 | 17.0 | 8.1 | 11.0 | NA |
| **Sandy clay** | 5.5 | NA | 3.6 | NA | -22.1 |
| **Loam** | 7.8 | 15.4 | 14.4 | NA | 19.6 |
| **Silt loam** | NA | 6.8 | -6.7 | NA | NA |
| **Clay loam** | 3.2 | 15.5 | 17.6 | 39.1 | 11.7 |
| **Silty clay loam** | NA | 11.8 | 47.2 | NA | 13.0 |
| **Silty clay** | NA | 4.8 | 1.1 | NA | NA |
| **Clay** | -0.4 | 20.3 | 26.5 | 62.8 | 50.9 |
| **Climate** |  |  |  |  |  |
| **Dry** | 9.5 | 16.3 | 13.4 | 23.5 | 1.5 |
| **Subtropical** | 3.1 | 5.1 | 5.5 | NA | 14.4 |
| **Temperate** | 4.9 | 15.5 | 22.1 | 41.1 | 25.3 |
| **Tropical** | 5.5 | 9.8 | 8.4 | NA | NA |
| **Production system** |  |  |  |  |  |
| **Greenhouse** | 12.6 | 21.2 | 22.8 | 41.6 | -6.0 |
| **Open-field** | 7.9 | 10.2 | 13.4 | 24.6 | 11.9 |
| * positive numbers indicate an increase and negative numbers indicate a decrease in WP.  NA data not available | | | | | |

Fig. S1-S5: Effect of various deficit irrigation levels on yield for individual studies and crop subgroups. The dotted line (effect size = 0) represents full irrigation. Effect size > 0 indicates increase and effect size < 0 indicates reduction in yield for deficit irrigation level relative to full irrigation. Non-overlapping confidence intervals (horizontal bars) indicate significant differences.

Fig. S6-S10: Effect of deficit irrigation levels on WP for individual studies and crop subgroups. The dotted line (effect size = 0) represents full irrigation. Effect size > 0 indicates increase and effect size <0 indicates reduction in WP for deficit irrigation level relative to full irrigation. Non-overlapping confidence intervals (horizontal bars) indicate significant differences

Fig S1: Yield >80%FI vs FI

Fig S1: continued

Fig S2: Yield 65-80%FI vs FI

Fig S2: Continued

Fig S3: Yield 50-65%FI vs FI

Fig S3: Continued

Fig S4: Yield 35-50%FI vs FI

Fig S5: Yield >35%FI vs FI

Fig S5: continued

Fig S6: WP >80%FI vs FI

Fig S7: WP 65-80%FI vs FI

Fig S8: WP 50-65%FI vs FI

Fig S9: WP 35-50%FI vs FI

Fig S10: WP <35%FI vs FI


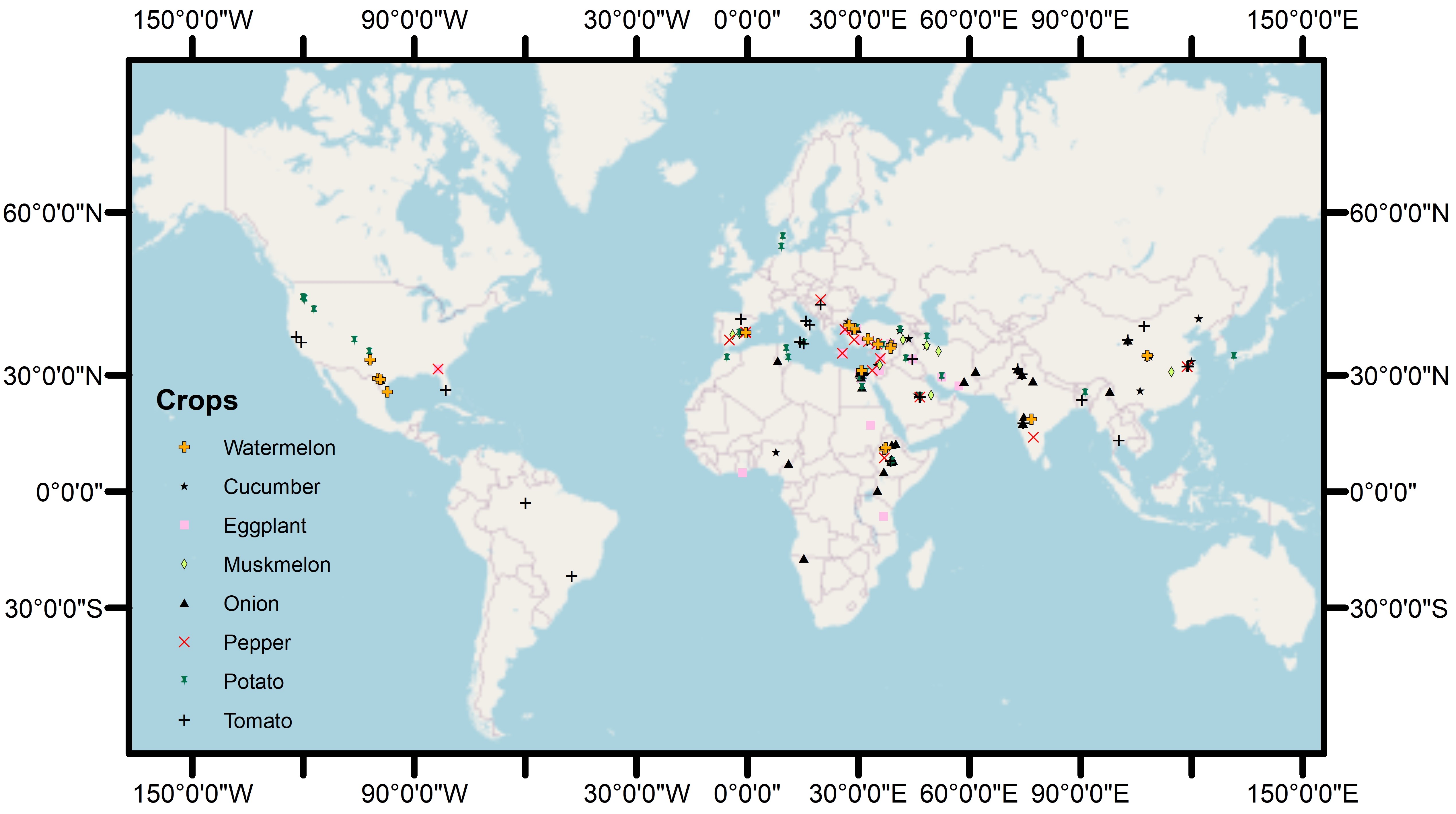


Fig S11: Locations of studies included in the meta-analysis. The map was created using ArcMAP version 10.8 (<https://www.esri.com/en-us/home>).

Table S4: PRISMA 2020 Checklist

| **Section and Topic** | **Item #** | **Checklist item** | **Location where item is reported** |
| --- | --- | --- | --- |
| **TITLE** | | |  |
| Title | 1 | Identify the report as a systematic review. | Page 1 |
| **ABSTRACT** | | |  |
| Abstract | 2 | See the PRISMA 2020 for Abstracts checklist. | Abstract checklist |
| **INTRODUCTION** | | |  |
| Rationale | 3 | Describe the rationale for the review in the context of existing knowledge. | Page 3-5 |
| Objectives | 4 | Provide an explicit statement of the objective(s) or question(s) the review addresses. | Page 5 |
| **METHODS** | | |  |
| Eligibility criteria | 5 | Specify the inclusion and exclusion criteria for the review and how studies were grouped for the syntheses. | Page 20 |
| Information sources | 6 | Specify all databases, registers, websites, organisations, reference lists and other sources searched or consulted to identify studies. Specify the date when each source was last searched or consulted. | Page 20 |
| Search strategy | 7 | Present the full search strategies for all databases, registers and websites, including any filters and limits used. | Page 20 |
| Selection process | 8 | Specify the methods used to decide whether a study met the inclusion criteria of the review, including how many reviewers screened each record and each report retrieved, whether they worked independently, and if applicable, details of automation tools used in the process. |  |
| Data collection process | 9 | Specify the methods used to collect data from reports, including how many reviewers collected data from each report, whether they worked independently, any processes for obtaining or confirming data from study investigators, and if applicable, details of automation tools used in the process. | Page 20-22 |
| Data items | 10a | List and define all outcomes for which data were sought. Specify whether all results that were compatible with each outcome domain in each study were sought (e.g. for all measures, time points, analyses), and if not, the methods used to decide which results to collect. | Page 21-22 |
|  | 10b | List and define all other variables for which data were sought (e.g. participant and intervention characteristics, funding sources). Describe any assumptions made about any missing or unclear information. | Page 21-22 |
| Study risk of bias assessment | 11 | Specify the methods used to assess risk of bias in the included studies, including details of the tool(s) used, how many reviewers assessed each study and whether they worked independently, and if applicable, details of automation tools used in the process. |  |
| Effect measures | 12 | Specify for each outcome the effect measure(s) (e.g. risk ratio, mean difference) used in the synthesis or presentation of results. | Page 23 |
| Synthesis methods | 13a | Describe the processes used to decide which studies were eligible for each synthesis (e.g. tabulating the study intervention characteristics and comparing against the planned groups for each synthesis (item #5)). | Page 22 and Table S1 |
|  | 13b | Describe any methods required to prepare the data for presentation or synthesis, such as handling of missing summary statistics, or data conversions. | Page 21-22 |
|  | 13c | Describe any methods used to tabulate or visually display results of individual studies and syntheses. | Page 23-24 |
|  | 13d | Describe any methods used to synthesize results and provide a rationale for the choice(s). If meta-analysis was performed, describe the model(s), method(s) to identify the presence and extent of statistical heterogeneity, and software package(s) used. | Page 23-24 |
|  | 13e | Describe any methods used to explore possible causes of heterogeneity among study results (e.g. subgroup analysis, meta-regression). | Page 23-24 |
|  | 13f | Describe any sensitivity analyses conducted to assess robustness of the synthesized results. | Page 23 |
| Reporting bias assessment | 14 | Describe any methods used to assess risk of bias due to missing results in a synthesis (arising from reporting biases). |  |
| Certainty assessment | 15 | Describe any methods used to assess certainty (or confidence) in the body of evidence for an outcome. |  |
| **RESULTS** | | |  |
| Study selection | 16a | Describe the results of the search and selection process, from the number of records identified in the search to the number of studies included in the review, ideally using a flow diagram. | Page 6-7 |
|  | 16b | Cite studies that might appear to meet the inclusion criteria, but which were excluded, and explain why they were excluded. |  |
| Study characteristics | 17 | Cite each included study and present its characteristics. | Table S1, Supplementary material. |
| Risk of bias in studies | 18 | Present assessments of risk of bias for each included study. |  |
| Results of individual studies | 19 | For all outcomes, present, for each study: (a) summary statistics for each group (where appropriate) and (b) an effect estimate and its precision (e.g. confidence/credible interval), ideally using structured tables or plots. | Fig. 1-5 and Fig. S1-S10 |
| Results of syntheses | 20a | For each synthesis, briefly summarise the characteristics and risk of bias among contributing studies. |  |
|  | 20b | Present results of all statistical syntheses conducted. If meta-analysis was done, present for each the summary estimate and its precision (e.g. confidence/credible interval) and measures of statistical heterogeneity. If comparing groups, describe the direction of the effect. | Fig. 1-5 and Fig. S1-S10 |
|  | 20c | Present results of all investigations of possible causes of heterogeneity among study results. | Section 2.2,2.3,2.4 and 2.5. Fig. 1-5 and Fig. S1-S10 |
|  | 20d | Present results of all sensitivity analyses conducted to assess the robustness of the synthesized results. |  |
| Reporting biases | 21 | Present assessments of risk of bias due to missing results (arising from reporting biases) for each synthesis assessed. |  |
| Certainty of evidence | 22 | Present assessments of certainty (or confidence) in the body of evidence for each outcome assessed. |  |
| **DISCUSSION** | | |  |
| Discussion | 23a | Provide a general interpretation of the results in the context of other evidence. | Page 11-17 |
|  | 23b | Discuss any limitations of the evidence included in the review. | Page 17-18 |
|  | 23c | Discuss any limitations of the review processes used. | Page 17-18 |
|  | 23d | Discuss implications of the results for practice, policy, and future research. | Page 17-20 |
| **OTHER INFORMATION** | | |  |
| Registration and protocol | 24a | Provide registration information for the review, including register name and registration number, or state that the review was not registered. | Not registered |
|  | 24b | Indicate where the review protocol can be accessed, or state that a protocol was not prepared. | Not registered |
|  | 24c | Describe and explain any amendments to information provided at registration or in the protocol. |  |
| Support | 25 | Describe sources of financial or non-financial support for the review, and the role of the funders or sponsors in the review. |  |
| Competing interests | 26 | Declare any competing interests of review authors. | Page 28 |
| Availability of data, code and other materials | 27 | Report which of the following are publicly available and where they can be found: template data collection forms; data extracted from included studies; data used for all analyses; analytic code; any other materials used in the review. | Page 24 |

Table S5: PRISMA Abstract Checklist

| **Topic** | **No.** | **Item** | **Reported?** |
| --- | --- | --- | --- |
| **TITLE** |  |  |  |
| **Title** | 1 | Identify the report as a systematic review. | Yes |
| **BACKGROUND** |  |  |  |
| **Objectives** | 2 | Provide an explicit statement of the main objective(s) or question(s) the review addresses. | Yes |
| **METHODS** |  |  |  |
| **Eligibility criteria** | 3 | Specify the inclusion and exclusion criteria for the review. | No |
| **Information sources** | 4 | Specify the information sources (e.g. databases, registers) used to identify studies and the date when each was last searched. | No |
| **Risk of bias** | 5 | Specify the methods used to assess risk of bias in the included studies. | No |
| **Synthesis of results** | 6 | Specify the methods used to present and synthesize results. | No |
| **RESULTS** |  |  |  |
| **Included studies** | 7 | Give the total number of included studies and participants and summarise relevant characteristics of studies. | Yes |
| **Synthesis of results** | 8 | Present results for main outcomes, preferably indicating the number of included studies and participants for each. If meta-analysis was done, report the summary estimate and confidence/credible interval. If comparing groups, indicate the direction of the effect (i.e. which group is favoured). | Yes |
| **DISCUSSION** |  |  |  |
| **Limitations of evidence** | 9 | Provide a brief summary of the limitations of the evidence included in the review (e.g. study risk of bias, inconsistency and imprecision). | No |
| **Interpretation** | 10 | Provide a general interpretation of the results and important implications. | Yes |
| **OTHER** |  |  |  |
| **Funding** | 11 | Specify the primary source of funding for the review. | No |
| **Registration** | 12 | Provide the register name and registration number. | No |

**References**

1 Şimşek, M., Kaçıra, M. & Tonkaz, T. The effects of different drip irrigation regimes on watermelon [Citrullus lanatus (Thunb.)] yield and yield components under semi-arid climatic conditions. *Australian Journal of Agricultural Research* **55**, doi:10.1071/ar03264 (2004).

2 Kirnak, H. & Demirtas, M. N. Effects of Different Irrigation Regimes and Mulches on Yield and Macronutrition Levels of Drip-Irrigated Cucumber Under Open Field Conditions. *Journal of Plant Nutrition* **29**, 1675-1690, doi:10.1080/01904160600851619 (2006).

3 Ertek, A., Şensoy, S., Gedik, İ. & Küçükyumuk, C. Irrigation scheduling based on pan evaporation values for cucumber (Cucumis sativus L.) grown under field conditions. *Agricultural Water Management* **81**, 159-172, doi:10.1016/j.agwat.2005.03.008 (2006).

4 Ayas, S. & Demirtas, C. Deficit irrigation effects on cucumber (Cucumis sativus L. Maraton) yield in unheated greenhouse condition. *Journal of Food, Agriculture & Environment* **7**, 645-649 (2009).

5 Wang, Z., Liu, Z., Zhang, Z. & Liu, X. Subsurface drip irrigation scheduling for cucumber (Cucumis sativus L.) grown in solar greenhouse based on 20cm standard pan evaporation in Northeast China. *Scientia Horticulturae* **123**, 51-57, doi:10.1016/j.scienta.2009.07.020 (2009).

6 Amer, K. H., Midan, S. A. & Hatfield, J. L. Effect of Deficit Irrigation and Fertilization on Cucumber. *Agronomy Journal* **101**, 1556-1564, doi:10.2134/agronj2009.0112 (2009).

7 Zhang, H.-x., Chi, D.-c., Wang, Q., Fang, J. & Fang, X.-y. Yield and Quality Response of Cucumber to Irrigation and Nitrogen Fertilization Under Subsurface Drip Irrigation in Solar Greenhouse. *Agricultural Sciences in China* **10**, 921-930, doi:<https://doi.org/10.1016/S1671-2927(11)60077-1> (2011).

8 Alomran, A., Louki, I., Aly, A. & Nadeem, M. Impact of deficit irrigation on soil salinity and cucumber yield under greenhouse condition in an arid environment. *Journal Of Agricultural Science And Technology* **15**, 1247-1259 (2013).

9 Rahil, M. H. & Qanadillo, A. Effects of different irrigation regimes on yield and water use efficiency of cucumber crop. *Agricultural Water Management* **148**, 10-15, doi:10.1016/j.agwat.2014.09.005 (2015).

10 Sahin, U., Kuslu, Y. & Kiziloglu, F. Response of cucumbers to different irrigation regimes applied through drip-irrigation system. *J. Anim. Plant Sci* **25**, 198-205 (2015).

11 Çakir, R., Kanburoglu-Çebi, U., Altintas, S. & Ozdemir, A. Irrigation scheduling and water use efficiency of cucumber grown as a spring-summer cycle crop in solar greenhouse. *Agricultural Water Management* **180**, 78-87, doi:10.1016/j.agwat.2016.10.023 (2017).

12 Abd El-Mageed, T. A., Semida, W. M., Taha, R. S. & Rady, M. M. Effect of summer-fall deficit irrigation on morpho-physiological, anatomical responses, fruit yield and water use efficiency of cucumber under salt affected soil. *Scientia Horticulturae* **237**, 148-155, doi:<https://doi.org/10.1016/j.scienta.2018.04.014> (2018).

13 Wang, A., Gallardo, M., Zhao, W., Zhang, Z. & Miao, M. Yield, nitrogen uptake and nitrogen leaching of tunnel greenhouse grown cucumber in a shallow groundwater region. *Agricultural Water Management* **217**, 73-80, doi:10.1016/j.agwat.2019.02.026 (2019).

14 Wang, H. *et al.* Optimal drip fertigation management improves yield, quality, water and nitrogen use efficiency of greenhouse cucumber. *Scientia Horticulturae* **243**, 357-366, doi:10.1016/j.scienta.2018.08.050 (2019).

15 Abdelraouf, R. E., Ghanem, H. G., A. Bukhari, N. & El-Zaidy, M. Field and Modeling Study on Manual and Automatic Irrigation Scheduling under Deficit Irrigation of Greenhouse Cucumber. *Sustainability* **12**, doi:10.3390/su12239819 (2020).

16 Ali, A. B., Elshaikh, N. A., Hussien, G., Abdallah, F. E. & Hassan, S. Biochar addition for enhanced cucumber fruit quality under deficit irrigation. *Bioscience Journal* **36**, doi:10.14393/BJ-v36n6a2020-47814 (2020).

17 Zakka, E. J., Onwuegbunam, N. E., Dare, A., Onwuegbunam, D. O. & Emeghara, U. U. Yield, water use and water productivity of drip-irrigated cucumber in response to irrigation depths and intervals in Kaduna, Nigeria. *Nigerian Journal of Technology* **39**, 613-620, doi:10.4314/njt.v39i2.33 (2020).

18 Ghahremani, Z., Mikaealzadeh, M., Barzegar, T. & Ranjbar, M. E. Foliar Application of Ascorbic Acid and Gamma Aminobutyric Acid Can Improve Important Properties of Deficit Irrigated Cucumber Plants (Cucumis sativus cv. Us). *Gesunde Pflanzen* **73**, 77-84, doi:10.1007/s10343-020-00530-6 (2020).

19 He, Z. *et al.* Determination of amount of irrigation and nitrogen for comprehensive growth of greenhouse cucumber based on multi-level fuzzy evaluation. *International Journal of Agricultural and Biological Engineering* **14**, 35-42, doi:10.25165/j.ijabe.20211402.5785 (2021).

20 Parkash, V., Singh, S., Deb, S. K., Ritchie, G. L. & Wallace, R. W. Effect of deficit irrigation on physiology, plant growth, and fruit yield of cucumber cultivars. *Plant Stress* **1**, doi:10.1016/j.stress.2021.100004 (2021).

21 Chartzoulakis, K. & Drosos, N. Water use and yield of greenhouse grown eggplant under drip irrigation. *Agricultural Water Management* **28**, 113-120, doi:<https://doi.org/10.1016/0378-3774(95)01173-G> (1995).

22 Kirnak, H., Tas, I., Kaya, C. & Higgs, D. Effects of deficit irrigation on growth, yield and fruit quality of eggplant under semi-arid conditions. *Australian Journal of Agricultural Research* **53**, 1367-1373, doi:<https://doi.org/10.1071/AR02014> (2002).

23 Senyigit, U., Kadayifci, A., Ozdemir, F. O., Oz, H. & Atilgan, A. Effects of different irrigation programs on yield and quality parameters of eggplant (Solanum melongena L.) under greenhouse conditions. *African Journal of Biotechnology* **10**, 6497-6503 (2011).

24 Demirel, K. *et al.* Yield estimate using spectral indices in eggplant and bell pepper grown under deficit irrigation. *Fresenius Environ. Bull* **23**, 1232-1237 (2014).

25 Díaz-Pérez, J. C. & Eaton, T. E. Eggplant (Solanum melongena L.) plant growth and fruit yield as affected by drip irrigation rate. *HortScience* **50**, 1709-1714 (2015).

26 Karajeh, M. & Mohawesh, O. Root-Knot Nematode (Meloidogyne javanica) – Deficit Irrigation Interactions on Eggplant Cropped under Open Field Conditions. *Journal of Horticultural Research* **24**, 73-78, doi:10.1515/johr-2016-0009 (2016).

27 Karam, F. & Nangia, V. Improving Water Productivity in Semi-arid Environments through Regulated Deficit Irrigation. *Annals of Arid Zone* **55**, 79-87 (2016).

28 Mohawesh, O. Utilizing Deficit Irrigation to Enhance Growth Performance and Water-use Efficiency of Eggplant in Arid Environments. *Journal of Agricultural Science and Technology* **18**, 265-276 (2016).

29 Çolak, Y. B., Yazar, A., Sesveren, S. & Çolak, İ. Evaluation of yield and leaf water potantial (LWP) for eggplant under varying irrigation regimes using surface and subsurface drip systems. *Scientia Horticulturae* **219**, 10-21, doi:10.1016/j.scienta.2017.02.051 (2017).

30 Abdrabbo, M., Saleh, S. & Hashem, F. Eggplant Production Under Deficit Irrigation and Polyethylene Mulch. *Egypt. J. of Appl. Sci* **32**, 2017 (2017).

31 Ayas, S. The Effects of Irrigation Regimes on the Yield and Water Use of Eggplant (Solanum melongena L.). *Toprak Su Dergisi* **6**, 49-58, doi:10.21657/topraksu.339835 (2017).

32 Darko, R. O., Yuan, S., Sekyere, J. D. O. & Liu, J. Effect of Deficit Irrigation on Yield and Quality of Eggplant. *International Journal of Environment, Agriculture and Biotechnology* **4**, 1325-1333, doi:10.22161/ijeab.45.5 (2019).

33 Bader, B. R., Abood, M. A., Aldulaimy, S. E. H., Al-Mehmdya, S. M. H. & Hamdi, G. J. Effect of water deficit and foliar application of amino acids on growth and yield of eggplant irrigated by two drip systems under greenhouse conditions. (2020).

34 Badr, M. A., El-Tohamy, W. A., Abou-Hussein, S. D. & Gruda, N. S. Deficit Irrigation and Arbuscular Mycorrhiza as a Water-Saving Strategy for Eggplant Production. *Horticulturae* **6**, doi:10.3390/horticulturae6030045 (2020).

35 Ibrahim, Y. M., Abdalla, M. M., Ali, O. O. & Abdalhi, M. Effect of irrigation systems and watering amount on yield of eggplant (Solanum melongena) under arid conditions. *Agricultural and Biological Sciences Journal* **6**, 143-147 (2020).

36 Abd El-Mageed, T. A., Abdelkhalik, A., Abd El-Mageed, S. A. & Semida, W. M. Co-composted Poultry Litter Biochar Enhanced Soil Quality and Eggplant Productivity Under Different Irrigation Regimes. *Journal of Soil Science and Plant Nutrition*, doi:10.1007/s42729-021-00490-4 (2021).

37 Ebrahimi, M., Souri, M. K., Mousavi, A. & Sahebani, N. Biochar and vermicompost improve growth and physiological traits of eggplant (Solanum melongena L.) under deficit irrigation. *Chemical and Biological Technologies in Agriculture* **8**, doi:10.1186/s40538-021-00216-9 (2021).

38 Mwinuka, P. R. *et al.* Optimizing water and nitrogen application for neglected horticultural species in tropical sub-humid climate areas: A case of African eggplant (Solanum aethiopicum L.). *Scientia Horticulturae* **276**, doi:10.1016/j.scienta.2020.109756 (2021).

39 Rodan, A. M., Hassandokht, M. R., Sadeghzadeh-Ahari, D. & Musavi, A. Alleviating of Water Deficit in Eggplant by Two Different Mulches. *Egyptian Journal of Horticulture* **48**, 37-48, doi:10.21608/ejoh.2020.53012.1155 (2021).

40 Semida, W. M. *et al.* Foliar Application of Zinc Oxide Nanoparticles Promotes Drought Stress Tolerance in Eggplant (Solanum melongena L.). *Plants (Basel)* **10**, doi:10.3390/plants10020421 (2021).

41 Fabeiro, C., Martı́n de Santa Olalla, F. & de Juan, J. A. Production of muskmelon (Cucumis melo L.) under controlled deficit irrigation in a semi-arid climate. *Agricultural Water Management* **54**, 93-105, doi:<https://doi.org/10.1016/S0378-3774(01)00151-2> (2002).

42 Kirnak, H., Higgs, D., Kaya, C. & Tas, I. Effects of Irrigation and Nitrogen Rates on Growth, Yield, and Quality of Muskmelon in Semiarid Regions. *Journal of Plant Nutrition* **28**, 621-638, doi:10.1081/pln-200052635 (2005).

43 Dogan, E., Kirnak, H., Berekatoglu, K., Bilgel, L. & Surucu, A. Water stress imposed on muskmelon (Cucumis Melo L.) with subsurface and surface drip irrigation systems under semi-arid climatic conditions. *Irrigation Science* **26**, 131-138, doi:10.1007/s00271-007-0079-7 (2007).

44 Sensoy, S., Ertek, A., Gedik, I. & Kucukyumuk, C. Irrigation frequency and amount affect yield and quality of field-grown melon (Cucumis melo L.). *Agricultural Water Management* **88**, 269-274, doi:10.1016/j.agwat.2006.10.015 (2007).

45 Cabello, M. J., Castellanos, M. T., Romojaro, F., Martínez-Madrid, C. & Ribas, F. Yield and quality of melon grown under different irrigation and nitrogen rates. *Agricultural Water Management* **96**, 866-874, doi:10.1016/j.agwat.2008.11.006 (2009).

46 Zeng, C.-Z., Bie, Z.-L. & Yuan, B.-Z. Determination of optimum irrigation water amount for drip-irrigated muskmelon (Cucumis melo L.) in plastic greenhouse. *Agricultural Water Management* **96**, 595-602, doi:10.1016/j.agwat.2008.09.019 (2009).

47 Al-Mefleh, N. K., Samarah, N., Zaitoun, S. & Al-Ghzawi, A. Effect of irrigation levels on fruit characteristics, total fruit yield and water use efficiency of melon under drip irrigation system. *Journal of Food, Agriculture & Environment* **10**, 540-545 (2012).

48 Ahmadi-Mirabad, A., Lotfi, M. & Roozban, M. R. Growth, yield, yield components and water-use efficiency in irrigated cantaloupes under full and deficit irrigation. *Electronic J Biol* **10**, 79-84 (2014).

49 Alenazi, M., Abdel-Razzak, H., Ibrahim, A., Wahb-Allah, M. & Alsadon, A. Response of muskmelon cultivars to plastic mulch and irrigation regimes under greenhouse conditions. *J. Anim. Plant Sci* **25**, 1398-1410 (2015).

50 Sharma, S. P., Leskovar, D. I., Crosby, K. C., Ibraim, A. M. H. & Volder, A. Differential response of muskmelon (Cucumis meloL.) cultivars to deficit irrigation. *Acta Horticulturae*, 201-208, doi:10.17660/ActaHortic.2016.1112.28 (2016).

51 Barzegar, T., Heidaryan, N., Lofti, H. & Ghahremani, Z. Yield, fruit quality and physiological responses of melon cv. Khatooni under deficit irrigation. *Advances in Horticultural Science* **32**, 451-458 (2018).

52 Akhoundnejad, Y. & Dasgan, H. Y. Photosynthesis, transpiration, stomatal conductance of some melon (Cucumic melo L.) genotypes under different drought stress. *Fresenius environmental bulletin* **29**, 10974-10979 (2020).

53 Ezzo, M. I., Mohamed, A. S., Glala, A. A. & Saleh, S. A. Utilization of grafting technique for sustaining cantaloupe productivity and quality under deficit irrigation water. *Bulletin of the National Research Centre* **44**, doi:10.1186/s42269-020-0283-7 (2020).

54 Yavuz, D., Seymen, M., Yavuz, N., Çoklar, H. & Ercan, M. Effects of water stress applied at various phenological stages on yield, quality, and water use efficiency of melon. *Agricultural Water Management* **246**, doi:10.1016/j.agwat.2020.106673 (2021).

55 Yavuz, N. Can grafting affect yield and water use efficiency of melon under different irrigation depths in a semi-arid zone? *Arabian Journal of Geosciences* **14**, doi:10.1007/s12517-021-07498-1 (2021).

56 Zeineldin, F. & Al-Molhim, Y. Polymer and deficit irrigation influence on water use efficiency and yield of muskmelon under surface and subsurface drip irrigation. *Soil and Water Research* **16**, 191-203, doi:10.17221/94/2020-swr (2021).

57 Bekele, S. & Tilahun, K. Regulated deficit irrigation scheduling of onion in a semiarid region of Ethiopia. *Agricultural Water Management* **89**, 148-152, doi:10.1016/j.agwat.2007.01.002 (2007).

58 Kumar, S., Imtiyaz, M., Kumar, A. & Singh, R. Response of onion (Allium cepa L.) to different levels of irrigation water. *Agricultural Water Management* **89**, 161-166, doi:10.1016/j.agwat.2007.01.003 (2007).

59 Patel, N. & Rajput, T. B. S. Effect of subsurface drip irrigation on onion yield. *Irrigation Science* **27**, 97-108, doi:10.1007/s00271-008-0125-0 (2008).

60 Ayas, S. & Demirtaș, Ç. Deficit irrigation effects on onion (Allium cepa L. E.T. Grano 502) yield in unheated greenhouse condition. *Journal of Food, Agriculture &amp; Environment* **7**, 239-243 (2009).

61 Enciso, J., Wiedenfeld, B., Jifon, J. & Nelson, S. Onion yield and quality response to two irrigation scheduling strategies. *Scientia Horticulturae* **120**, 301-305, doi:10.1016/j.scienta.2008.11.004 (2009).

62 Nagaz, K., Masmoudi, M. M. & Ben Mechlia, N. Yield Response of Drip-Irrigated Onion under Full and Deficit Irrigation with Saline Water in Arid Regions of Tunisia. *ISRN Agronomy* **2012**, 1-8, doi:10.5402/2012/562315 (2012).

63 Igbadun, H. E., Ramalan, A. A. & Oiganji, E. Effects of regulated deficit irrigation and mulch on yield, water use and crop water productivity of onion in Samaru, Nigeria. *Agricultural Water Management* **109**, 162-169, doi:10.1016/j.agwat.2012.03.006 (2012).

64 Leskovar, D. I., Agehara, S., Yoo, K. & Pascual-Seva, N. Crop coefficient-based deficit irrigation and planting density for onion: growth, yield, and bulb quality. *HortScience* **47**, 31-37 (2012).

65 Zheng, J. *et al.* Effects of water deficits on growth, yield and water productivity of drip-irrigated onion (Allium cepa L.) in an arid region of Northwest China. *Irrigation Science* **31**, 995-1008, doi:10.1007/s00271-012-0378-5 (2012).

66 Patel, N. & Rajput, T. Effect of deficit irrigation on crop growth, yield and quality of onion in subsurface drip irrigation. *International Journal of Plant Production* **7**, 417-435 (2013).

67 Rop, D. K., Kipkorir, E. C. & Taragon, J. K. Effects of Deficit Irrigation on Yield and Quality of Onion Crop. *Journal of Agricultural Science* **8**, doi:10.5539/jas.v8n3p112 (2016).

68 Enchalew, B. *et al.* Effect of deficit irrigation on water productivity of onion (Allium cepal.) under drip irrigation. *Irrigat Drainage Sys Eng* **5**, 2 (2016).

69 Wakchaure, G. C. *et al.* Growth, bulb yield, water productivity and quality of onion (Allium cepa L.) as affected by deficit irrigation regimes and exogenous application of plant bio–regulators. *Agricultural Water Management* **199**, 1-10, doi:10.1016/j.agwat.2017.11.026 (2018).

70 Afzal, M., Cheema, M., Shahid, M., Arshad, M. & Khaliq, T. Optimization of subsurface drip lateral depths and irrigation levels for best yield response of onion (Allium cepa L.). *J. Anim. Plant Sci* **30**, 702-712 (2020).

71 Ambomsa, A. & Seyoum, D. *Effect of irrigation methods and irrigation levels on yield and water productivity of onion at Awash Melkasa, Ethiopia*, Haramaya university, (2019).

72 Dingre, S. & Pawar, D. Response of drip irrigated onion (Allium cepa L.) growth, yield and water productivity under deficit irrigation schedules. *Journal of Natural Resource Conservation and Management* **1**, 69-75 (2020).

73 Hefzy, M., Mostafa, H. & Zahran, M. Onion drought tolerance enhancement in calcareous soils based on using bio-stimulants. *Environment, Biodiversity and Soil Security* **0**, 0-0, doi:10.21608/jenvbs.2020.41196.1104 (2020).

74 Kandongo, H., Mudamburi, B., Akundabweni, L., Hove, K. & Hatutale, G. Effect of Deficit Irrigation and Irrigarion Types on Yield and Gross Margin of Onion in a Semi-Arid Sub-Saharean Africa Namibia, Omusati Region. *International Journal of Recent Engineering Research and Development* **5** (2020).

75 Mugoro, T., Assefa, S. & Getahun, A. Effect of deficit irrigation on yield and water productivity of onion (Allium cepa L.) under conventional furrow irrigation system, in Bennatsemay Woreda, Southern Ethiopia. *J. Agric. Biol. Eng.* **181**, 2-13 (2020).

76 Nurga, Y., Alemayehu, Y. & Abegaz, F. Effect of deficit irrigation levels at different growth stages on yield and water productivity of onion (Allium cepa L.) at Raya Azebo Woreda, Northern Ethiopia. *Ethiopian Journal of Agricultural Sciences* **30**, 155-176 (2020).

77 Piri, H. & Naserin, A. Effect of different levels of water, applied nitrogen and irrigation methods on yield, yield components and IWUE of onion. *Scientia Horticulturae* **268**, doi:10.1016/j.scienta.2020.109361 (2020).

78 Semida, W. M., Abdelkhalik, A., Rady, M. O. A., Marey, R. A. & Abd El-Mageed, T. A. Exogenously applied proline enhances growth and productivity of drought stressed onion by improving photosynthetic efficiency, water use efficiency and up-regulating osmoprotectants. *Scientia Horticulturae* **272**, doi:10.1016/j.scienta.2020.109580 (2020).

79 Shirzadi, M. H., Arvin, M. J., Abootalebi, A., Hasandokht, M. R. & Tejada Moral, M. Effect of nylon mulch and some plant growth regulators on water use efficiency and some quantitative traits in onion (Allium cepa cv.) under water deficit stress. *Cogent Food & Agriculture* **6**, doi:10.1080/23311932.2020.1779562 (2020).

80 El–Metwally, I., Geries, L. & Saudy, H. Interactive effect of soil mulching and irrigation regime on yield, irrigation water use efficiency and ‎weeds of trickle–irrigated onion. *Archives of Agronomy and Soil Science*, 1-13, doi:10.1080/03650340.2020.1869723 (2021).

81 Tegenu, G. Regulated Deficit Irrigation and Onion (Allium cepa L.) Yield Response on Water Productivity. *Irrigation and Drainage*

*Systems Engineering* **10** (2021).

82 Wakchaure, G. C. *et al.* Quantification of water stress impacts on canopy traits, yield, quality and water productivity of onion (Allium cepa L.) cultivars in a shallow basaltic soil of water scarce zone. *Agricultural Water Management* **249**, doi:10.1016/j.agwat.2021.106824 (2021).

83 Chartzoulakis, K. & Drosos, N. Water Requirements of Greenhouse Grown Pepper under Drip Irrigation. *Acta Horticulturae*, 175-180, doi:10.17660/ActaHortic.1997.449.23 (1997).

84 González-Dugo, V., Orgaz, F. & Fereres, E. Responses of pepper to deficit irrigation for paprika production. *Scientia Horticulturae* **114**, 77-82, doi:10.1016/j.scienta.2007.05.014 (2007).

85 Guang-Cheng, S., Zhang, Z.-Y., Liu, N., Yu, S.-E. & Xing, W.-G. Comparative effects of deficit irrigation (DI) and partial rootzone drying (PRD) on soil water distribution, water use, growth and yield in greenhouse grown hot pepper. *Scientia Horticulturae* **119**, 11-16, doi:10.1016/j.scienta.2008.07.001 (2008).

86 Gadissa, T. & Chemeda, D. Effects of drip irrigation levels and planting methods on yield and yield components of green pepper (Capsicum annuum, L.) in Bako, Ethiopia. *Agricultural Water Management* **96**, 1673-1678, doi:10.1016/j.agwat.2009.07.004 (2009).

87 Karam, F., Masaad, R., Bachour, R., Rhayem, C. & Rouphael, Y. Water and radiation use efficiencies in drip-irrigated pepper (Capsicum annuum L.): response to full and deficit irrigation regimes. *European Journal of Horticultural Science* **74**, 79-85 (2009).

88 Guang-Cheng, S., Na, L., Zhan-Yu, Z., Shuang-En, Y. & Chang-ren, C. Growth, yield and water use efficiency response of greenhouse-grown hot pepper under Time-Space deficit irrigation. *Scientia Horticulturae* **126**, 172-179, doi:10.1016/j.scienta.2010.07.003 (2010).

89 AlHarbi, A., Saleh, A., Al-Omran, A. & Wahb-Allah, M. in *International Symposium on Growing Media and Soilless Cultivation 1034.* 443-450.

90 Ćosić, M. *et al.* Effect of irrigation regime and application of kaolin on yield, quality and water use efficiency of sweet pepper. *Agricultural Water Management* **159**, 139-147, doi:10.1016/j.agwat.2015.05.014 (2015).

91 Kuşçu, H., Turhan, A., Özmen, N., Aydınol, P. & Demir, A. O. Response of red pepper to deficit irrigation and nitrogen fertigation. *Archives of Agronomy and Soil Science* **62**, 1396-1410, doi:10.1080/03650340.2016.1149818 (2016).

92 Celebi, M. The effects of water stress on yield performance of drip-irrigated pepper (Capsicum annum L. cv. Capya var. Yalova yağlık 28) in the Central Anatolian region of Turkey. *Arabian Journal of Geosciences* **11**, doi:10.1007/s12517-018-4086-1 (2018).

93 Sezen, S. M., Yazar, A. & Tekin, S. Physiological response of red pepper to different irrigation regimes under drip irrigation in the Mediterranean region of Turkey. *Scientia Horticulturae* **245**, 280-288, doi:10.1016/j.scienta.2018.10.037 (2019).

94 Abdelkhalik, A. *et al.* Effects of deficit irrigation on the yield and irrigation water use efficiency of drip-irrigated sweet pepper (Capsicum annuum L.) under Mediterranean conditions. *Irrigation Science* **38**, 89-104, doi:10.1007/s00271-019-00655-1 (2019).

95 Badawi, T., El-Kassas, M., Mahmoud, M. & ElKasas, A. Effect of Irrigation Levels and Soil Amendment on Growth and Yield of Sweet Pepper Crop under El-Arish Region Conditions. *Sinai Journal of Applied Sciences* **0**, 0-0, doi:10.21608/sinjas.2020.86367 (2020).

96 Demir, Z. & Özbahçe, A. Evaluating the effects of different irrigation and nitrogen applications on soil water content and yield quality parameters of pepper using surface and subsurface drip irrigation

*. *Irrigation and Drainage*, doi:10.1002/ird.2604 (2021).

97 Gisbert-Mullor, R. *et al.* Grafting onto an Appropriate Rootstock Reduces the Impact on Yield and Quality of Controlled Deficit Irrigated Pepper Crops. *Agronomy* **10**, doi:10.3390/agronomy10101529 (2020).

98 Sumathi, P., Kumar, H. V. H., Krishna, G. M. & Raju, J. T. Influence of Deficit Irrigation on Biometric Parameters of Capsicum Crop under Polyhouse Conditions. *International Research Journal of Pure and Applied Chemistry*, 341-347, doi:10.9734/irjpac/2020/v21i2430375 (2020).

99 Kabir, M. Y., Nambeesan, S. U., Bautista, J. & Díaz-Pérez, J. C. Effect of irrigation level on plant growth, physiology and fruit yield and quality in bell pepper (Capsicum annuum L.). *Scientia Horticulturae* **281**, doi:10.1016/j.scienta.2021.109902 (2021).

100 Martin, M. W. & Miller, D. E. Variations in responses of potato germplasm to deficit irrigation as affected by soil texture. *American Potato Journal* **60**, 671-683, doi:10.1007/BF02852838 (1983).

101 Shock, C. C., Feibert, E. B. G. & Saunders, L. D. Potato Yield and Quality Response to Deficit Irrigation. *HortScience* **33**, 655-659, doi:10.21273/hortsci.33.4.655 (1998).

102 Fabeiro, C., Martı́n de Santa Olalla, F. & de Juan, J. A. Yield and size of deficit irrigated potatoes. *Agricultural Water Management* **48**, 255-266, doi:<https://doi.org/10.1016/S0378-3774(00)00129-3> (2001).

103 Alva, A. K., Hodges, T., Boydston, R. A. & Collins, H. P. Effects of irrigation and tillage practices on yield of potato under high production conditions in the Pacific Northwest. *Communications in Soil Science and Plant Analysis* **33**, 1451-1460, doi:<https://doi.org/10.1081/CSS-120004293> (2002).

104 Yuan, B.-Z., Nishiyama, S. & Kang, Y. Effects of different irrigation regimes on the growth and yield of drip-irrigated potato. *Agricultural Water Management* **63**, 153-167, doi:10.1016/s0378-3774(03)00174-4 (2003).

105 Onder, S., Caliskan, M. E., Onder, D. & Caliskan, S. Different irrigation methods and water stress effects on potato yield and yield components. *Agricultural Water Management* **73**, 73-86, doi:10.1016/j.agwat.2004.09.023 (2005).

106 Kiziloglu, F. M., Sahin, U., Tunc, T. & Diler, S. The Effect of Deficit Irrigation on Potato Evapotranspiration and Tuber Yield under Cool Season and Semiarid Climatic Conditions. *Journal of Agronomy* **5**, 284-288, doi:10.3923/ja.2006.284.288 (2006).

107 Ierna, A. & Mauromicale, G. Physiological and growth response to moderate water deficit of off-season potatoes in a Mediterranean environment. *Agricultural Water Management* **82**, 193-209, doi:10.1016/j.agwat.2005.05.005 (2006).

108 Shahnazari, A. *et al.* Nitrogen dynamics in the soil-plant system under deficit and partial root-zone drying irrigation strategies in potatoes. *European Journal of Agronomy* **28**, 65-73, doi:10.1016/j.eja.2007.05.003 (2008).

109 Alva, A., Collins, H., Boydston, R. & Moore, A. in *International Crop Science Congress Proceedings.*

110 Ahmadi, S. H. *et al.* Effects of irrigation strategies and soils on field grown potatoes: Yield and water productivity. *Agricultural Water Management* **97**, 1923-1930, doi:<https://doi.org/10.1016/j.agwat.2010.07.007> (2010).

111 Badr, M. A., Abou Hussein, S. D., El-Tohamy, W. A. & Gruda, N. Efficiency of Subsurface Drip Irrigation for Potato Production Under Different Dry Stress Conditions. *Gesunde Pflanzen* **62**, 63-70, doi:10.1007/s10343-010-0222-x (2010).

112 Ayas, S. & Korukçu, A. Water-yield relationships in deficit irrigated potato. *J. of Agric. Fac. of Uludag Univ* **24**, 23-36 (2010).

113 Badr, M. A., El-Tohamy, W. A. & Zaghloul, A. M. Yield and water use efficiency of potato grown under different irrigation and nitrogen levels in an arid region. *Agricultural Water Management* **110**, 9-15, doi:10.1016/j.agwat.2012.03.008 (2012).

114 Ierna, A. & Mauromicale, G. Tuber yield and irrigation water productivity in early potatoes as affected by irrigation regime. *Agricultural Water Management* **115**, 276-284, doi:10.1016/j.agwat.2012.09.011 (2012).

115 Ahmadi, S. H., Agharezaee, M., Kamgar-Haghighi, A. A. & Sepaskhah, A. R. Effects of dynamic and static deficit and partial root zone drying irrigation strategies on yield, tuber sizes distribution, and water productivity of two field grown potato cultivars. *Agricultural Water Management* **134**, 126-136, doi:<https://doi.org/10.1016/j.agwat.2013.11.015> (2014).

116 Maralian, H., Nasrollahzadeh, S., Raiyi, Y. & Hassanpanah, D. Responses of potato genotypes to limited irrigation. *IJAAR* **5**, 13-19 (2014).

117 Ghazouani, H. *et al.* Effect of different saline Levels and Irrigation Regimes on agronomic parameters of potatoes crop under the semi-arid environment of Tunisia. *Scientia Agriculturae* **12**, 99-104 (2015).

118 Mokh, F. E., Nagaz, K., Masmoudi, M. M. & Mechlia, N. B. Yield and Water Productivity of Drip-Irrigated Potato under Different Nitrogen Levels and Irrigation Regime with Saline Water in Arid Tunisia. *American Journal of Plant Sciences* **06**, 501-510, doi:10.4236/ajps.2015.64054 (2015).

119 Nouri, A., Nezami, A., Kafi, M. & Hassanpanah, D. Growth and yield response of potato genotypes to deficit irrigation. *International Journal of Plant Production* **10**, 139-157, doi:10.22069/ijpp.2016.2785 (2016).

120 Ghazouani, H. *et al.* Combining Fao-56 with an Economic Model to Assess Deficit Irrigation of Moderate Sensible Crop for Water Stress under Semi Arid Climate. *Current Politics and Economics of Africa* **10**, 1-9 (2017).

121 Zin El-Abedin, T. K., Mattar, M. A., Alazba, A. A. & Al-Ghobari, H. M. Comparative effects of two water-saving irrigation techniques on soil water status, yield, and water use efficiency in potato. *Scientia Horticulturae* **225**, 525-532, doi:10.1016/j.scienta.2017.07.044 (2017).

122 Barakat, M. A. S., Abd El-Mageed, T. A., Elsayed, I. N. & Semida, W. M. Effect of soil mulching on growth, productivity, and water use efficiency of potato (Solanum tuberosum L.) under deficit irrigation. *Archives of Agriculture and Environmental Science* **5**, 328-336, doi:10.26832/24566632.2020.0503014 (2020).

123 El Youssfi, L. *et al.* Agro-physiological response of potato to “sustainable” deficit irrigation in the plain of Saïs, Morocco. *E3S Web of Conferences* **183**, doi:10.1051/e3sconf/202018303001 (2020).

124 Elmetwalli, A. H. & Elnemr, M. K. Influence of deficit irrigation and nitrogen fertilization on potato yield, water productivity and net profit. *Agricultural Engineering International: CIGR Journal* **22**, 61-68 (2020).

125 Abd El-Wahed, M. H. *et al.* Salt Distribution and Potato Response to Irrigation Regimes under Varying Mulching Materials. *Plants (Basel)* **9**, doi:10.3390/plants9060701 (2020).

126 Essah, S. Y. C., Andales, A. A., Bauder, T. A. & Holm, D. G. Response of Two Colorado Russet Potato Cultivars to Reduced Irrigation Water Use. *American Journal of Potato Research* **97**, 221-233, doi:10.1007/s12230-020-09771-7 (2020).

127 Gogoi, M., Ray, L. I. P., Swami, S., Kant, K. & Meena, N. K. Performance of potato variety Kufri Megha under different irrigation scheduling and date of planting at North Eastern Indian mid hills. *Journal of Environmental Biology* **41**, 1605-1610, doi:10.22438/jeb/41/6/SI-225 (2020).

128 Kassaye, K. T., Yilma, W. A., Fisha, M. H., Haile, D. H. & Wu, W. Yield and Water Use Efficiency of Potato under Alternate Furrows and Deficit Irrigation. *International Journal of Agronomy* **2020**, 1-11, doi:10.1155/2020/8869098 (2020).

129 Mattar, M. A., Zin El-Abedin, T. K., Al-Ghobari, H. M., Alazba, A. A. & Elansary, H. O. Effects of different surface and subsurface drip irrigation levels on growth traits, tuber yield, and irrigation water use efficiency of potato crop. *Irrigation Science* **39**, 517-533, doi:10.1007/s00271-020-00715-x (2021).

130 O’Shaughnessy, S. A. *et al.* Irrigation Management of Potatoes Using Sensor Feedback: Texas High Plains. *Transactions of the ASABE* **63**, 1259-1276, doi:10.13031/trans.13925 (2020).

131 Zahran, M. M., Hefzy, M. & Mostafa, H. H. Enhancing the productivity of potato crop under drought stress by using some biological treatments. *Int. J. Environ* **9**, 83-103 (2020).

132 Al-Shamary, W. F. A., Alkhateb, B. A., Ghani, E. T. A., Al-Antary, T. M. & Kahlel, A.-M. S. The influence of perlite and irrigation management on the properties of potatoes in gypsiferous soil. *Fresenius Environmental Bulletin* **30**, 4771-4778 (2021).

133 Obreza, T. A., Pitts, D. J., McGovern, R. J. & Spreen, T. H. Deficit Irrigation of Micro-Irrigated Tomato Affects Yield, Fruit Quality, and Disease Severity. *Journal of Production Agriculture* **9**, 270-275, doi:10.2134/jpa1996.0270 (1996).

134 Kirda, C. *et al.* Yield response of greenhouse grown tomato to partial root drying and conventional deficit irrigation. *Agricultural Water Management* **69**, 191-201, doi:10.1016/j.agwat.2004.04.008 (2004).

135 Harmanto, Salokhe, V. M., Babel, M. S. & Tantau, H. J. Water requirement of drip irrigated tomatoes grown in greenhouse in tropical environment. *Agricultural Water Management* **71**, 225-242, doi:10.1016/j.agwat.2004.09.003 (2005).

136 del Amor, M. A. & del Amor, F. M. Response of tomato plants to deficit irrigation under surface or subsurface drip irrigation. *Journal of Applied Horticulture* **9**, 97-100 (2007).

137 Singh, R., Kumar, S., Nangare, D. & Meena, M. Drip irrigation and black polyethylene mulch influence on growth, yield and water-use efficiency of tomato. *African Journal of Agricultural Research* **4**, 1427-1430 (2009).

138 Patanè, C. & Cosentino, S. L. Effects of soil water deficit on yield and quality of processing tomato under a Mediterranean climate. *Agricultural Water Management* **97**, 131-138, doi:10.1016/j.agwat.2009.08.021 (2010).

139 Ozbahce, A. & Tari, A. F. Effects of different emitter space and water stress on yield and quality of processing tomato under semi-arid climate conditions. *Agricultural Water Management* **97**, 1405-1410, doi:10.1016/j.agwat.2010.04.008 (2010).

140 Wahb-Allah, M. A., Alsadon, A. A. & Ibrahim, A. A. Drought tolerance of several tomato genotypes under greenhouse conditions. *World Applied Sciences Journal* **15**, 933-940 (2011).

141 Patanè, C., Tringali, S. & Sortino, O. Effects of deficit irrigation on biomass, yield, water productivity and fruit quality of processing tomato under semi-arid Mediterranean climate conditions. *Scientia Horticulturae* **129**, 590-596, doi:10.1016/j.scienta.2011.04.030 (2011).

142 Mahadeen, A. Y., Mohawesh, O. E., Al-Absi, K. & Al-Shareef, W. Effect of irrigation regimes on water use efficiency and tomato yield (Lycopersicon esculentumMill.) grown in an arid environment. *Archives of Agronomy and Soil Science* **57**, 105-114, doi:10.1080/03650340903225024 (2011).

143 Hassan, A. M. & Abuarab, M. E. Effect of deficit irrigation water on the productivity and characteristics of tomato. *Misr J. Ag. Eng* **30**, 403-424 (2013).

144 Kuşçu, H., Turhan, A. & Demir, A. O. The response of processing tomato to deficit irrigation at various phenological stages in a sub-humid environment. *Agricultural Water Management* **133**, 92-103, doi:10.1016/j.agwat.2013.11.008 (2014).

145 Wahb-Allah, M., Abdel-Razzak, H., Alsadon, A. & Ibrahim, A. Growth, yield, fruit quality and water use efficiency of tomato under arbuscular mycorrhizal inoculation and irrigation level treatments. *Life Sci. J* **11**, 109-117 (2014).

146 Ibrahim, A., Wahb-Allah, M., Abdel-Razzak, H. & Alsadon, A. Growth, yield, quality and water use efficiency of grafted tomato plants grown in greenhouse under different irrigation levels. *Life Sci. J* **11**, 118-126 (2014).

147 Biswas, S., Akanda, A., Rahman, M. & Hossain, M. Effect of drip irrigation and mulching on yield, water-use efficiency and economics of tomato. *Plant, Soil and Environment* **61**, 97-102 (2015).

148 Kumar, P. S. *et al.* Influence of growth stage specific water stress on the yield, physico-chemical quality and functional characteristics of tomato grown in shallow basaltic soils. *Scientia Horticulturae* **197**, 261-271, doi:10.1016/j.scienta.2015.09.054 (2015).

149 Wang, C. *et al.* Assessing the response of yield and comprehensive fruit quality of tomato grown in greenhouse to deficit irrigation and nitrogen application strategies. *Agricultural Water Management* **161**, 9-19, doi:10.1016/j.agwat.2015.07.010 (2015).

150 Lahoz, I. *et al.* Effect of water deficit on the agronomical performance and quality of processing tomato. *Scientia Horticulturae* **200**, 55-65, doi:10.1016/j.scienta.2015.12.051 (2016).

151 Nangare, D. D., Singh, Y., Kumar, P. S. & Minhas, P. S. Growth, fruit yield and quality of tomato (Lycopersicon esculentum Mill.) as affected by deficit irrigation regulated on phenological basis. *Agricultural Water Management* **171**, 73-79, doi:10.1016/j.agwat.2016.03.016 (2016).

152 Cantore, V. *et al.* Combined effect of deficit irrigation and strobilurin application on yield, fruit quality and water use efficiency of “cherry” tomato (Solanum lycopersicum L.). *Agricultural Water Management* **167**, 53-61, doi:10.1016/j.agwat.2015.12.024 (2016).

153 Bowles, T. M., Barrios-Masias, F. H., Carlisle, E. A., Cavagnaro, T. R. & Jackson, L. E. Effects of arbuscular mycorrhizae on tomato yield, nutrient uptake, water relations, and soil carbon dynamics under deficit irrigation in field conditions. *Sci Total Environ* **566-567**, 1223-1234, doi:10.1016/j.scitotenv.2016.05.178 (2016).

154 Djurović, N., Ćosić, M., Stričević, R., Savić, S. & Domazet, M. Effect of irrigation regime and application of kaolin on yield, quality and water use efficiency of tomato. *Scientia Horticulturae* **201**, 271-278, doi:10.1016/j.scienta.2016.02.017 (2016).

155 Xiukang, W. & Yingying, X. Evaluation of the Effect of Irrigation and Fertilization by Drip Fertigation on Tomato Yield and Water Use Efficiency in Greenhouse. *International Journal of Agronomy* **2016**, 1-10, doi:10.1155/2016/3961903 (2016).

156 Du, Y.-d., Cao, H.-x., Liu, S.-q., Gu, X.-b. & Cao, Y.-x. Response of yield, quality, water and nitrogen use efficiency of tomato to different levels of water and nitrogen under drip irrigation in Northwestern China. *Journal of Integrative Agriculture* **16**, 1153-1161, doi:10.1016/s2095-3119(16)61371-0 (2017).

157 Wang, X. & Xing, Y. Evaluation of the effects of irrigation and fertilization on tomato fruit yield and quality: a principal component analysis. *Sci Rep* **7**, 350, doi:10.1038/s41598-017-00373-8 (2017).

158 Agbna, G. H. D. *et al.* Effects of deficit irrigation and biochar addition on the growth, yield, and quality of tomato. *Scientia Horticulturae* **222**, 90-101, doi:<https://doi.org/10.1016/j.scienta.2017.05.004> (2017).

159 Giuliani, M., Nardella, E., Gagliardi, A. & Gatta, G. Deficit Irrigation and Partial Root-Zone Drying Techniques in Processing Tomato Cultivated under Mediterranean Climate Conditions. *Sustainability* **9**, doi:10.3390/su9122197 (2017).

160 Abdelhady, S. A., El-Azm, N. A. & El-Kafafi, E.-S. H. Effect of deficit irrigation levels and NPK fertilization rates on tomato growth, yield and fruits quality. *Middle East Journal of Agriculture Research* **6**, 587-604 (2017).

161 Zhang, H., Xiong, Y., Huang, G., Xu, X. & Huang, Q. Effects of water stress on processing tomatoes yield, quality and water use efficiency with plastic mulched drip irrigation in sandy soil of the Hetao Irrigation District. *Agricultural Water Management* **179**, 205-214, doi:10.1016/j.agwat.2016.07.022 (2017).

162 Hashem, S. M., Zin El-Abedin, T. & M Al-Ghobari, H. Assessing effects of deficit irrigation techniques on water productivity of tomato for subsurface drip irrigation system. *International Journal of Agricultural and Biological Engineering* **11**, 137-145, doi:10.25165/j.ijabe.20181104.3846 (2018).

163 Al-Shmmari, A., Abood, M. & Hamdi, G. Improvement in Production, Fruit Quality and Water Use Efficiency of Three Tomato Cultivars by Foliar Application of Tecamin Flower® Under Water Deficit Conditions. *Journal of Central European Agriculture* **21**, 379-385, doi:10.5513/jcea01/21.2.2604 (2020).

164 Abd El- Aziz, M. The effect of Si on minimizing the implications of water stress on tomato plants. *Environment, Biodiversity and Soil Security* **0**, 0-0, doi:10.21608/jenvbs.2020.28732.1092 (2020).

165 Kumsa, L. T. Effect of Deficit Furrow Irrigation on Yield and Water Productivity of Tomato (Solanum Lycopersicum L.) in Central Rift Valley Intensive Irrigation System at East Shewa Zone, Oromia, Ethiopia. *International Journal of Engineering Research & Technology* **9**, 323-333 (2020).

166 Mattar, M. A., Zin El-Abedin, T. K., Alazba, A. A. & Al-Ghobari, H. M. Soil water status and growth of tomato with partial root-zone drying and deficit drip irrigation techniques. *Irrigation Science* **38**, 163-176, doi:10.1007/s00271-019-00658-y (2019).

167 Mendonça, T. G., Silva, M. B. d., Pires, R. C. d. M. & Souza, C. F. Deficit Irrigation of Subsurface Drip-Irrigated Grape Tomato. *Engenharia Agrícola* **40**, 453-461, doi:10.1590/1809-4430-eng.agric.v40n4p453-461/2020 (2020).

168 Milkereit, J., Stoddard, C. S., Dito, D. F. & Hodson, A. K. The influence of leaf traits and deficit irrigation on insect communities in mature green tomato production. *Agricultural and Forest Entomology* **22**, 119-128, doi:10.1111/afe.12365 (2019).

169 Patanè, C., Corinzia, S. A., Testa, G., Scordia, D. & Cosentino, S. L. Physiological and Agronomic Responses of Processing Tomatoes to Deficit Irrigation at Critical Stages in a Semi-Arid Environment. *Agronomy* **10**, doi:10.3390/agronomy10060800 (2020).

170 Shabbir, A. *et al.* Effects of Drip Irrigation Emitter Density with Various Irrigation Levels on Physiological Parameters, Root, Yield, and Quality of Cherry Tomato. *Agronomy* **10**, doi:10.3390/agronomy10111685 (2020).

171 Zahid, B., Ansari, R., Cheema, M. & Anjum, L. Evaluation of deficit irrigation regime, row spacing and dual plantation of drip irrigated tomato under high tunnel. *Journal of Central European Agriculture* **21**, 851-860, doi:10.5513/jcea01/21.4.2990 (2020).

172 Wu, Y. *et al.* Responses of growth, fruit yield, quality and water productivity of greenhouse tomato to deficit drip irrigation. *Scientia Horticulturae* **275**, doi:10.1016/j.scienta.2020.109710 (2021).

173 Erdem, Y., Yüksel, A. N. & Orta, A. H. The effects of deficit irrigation on watermelon yield, water use and quality characteristics. *Pakistan Journal of Biological Sciences* **4**, 785-789 (2001).

174 Leskovar, D., Bang, H., Kolenda, K., Franco, J. & Perkins-Veazie, P. in *XXVI International Horticultural Congress: Issues and Advances in Postharvest Horticulture 628.* 147-151.

175 Bang, H., Leskovar, D. I., Bender, D. A. & Crosby, K. Deficit irrigation impact on lycopene, soluble solids, firmness and yield of diploid and triploid watermelon in three distinct environments. *The Journal of Horticultural Science and Biotechnology* **79**, 885-890, doi:10.1080/14620316.2004.11511861 (2004).

176 Kirnak, H., Doğan, E., Bilgel, L. & Berakatoğlu, K. Effect of Preharvest Deficit Irrigation on Second Crop Watermelon Grown in an Extremely Hot Climate. *Journal of Irrigation and Drainage Engineering* **135**, 141-148, doi:doi:10.1061/(ASCE)0733-9437(2009)135:2(141) (2009).

177 Özmen, S., Kanber, R., Sarı, N. & Ünlü, M. The effects of deficit irrigation on nitrogen consumption, yield, and quality in drip irrigated grafted and ungrafted watermelon. *Journal of Integrative Agriculture* **14**, 966-976, doi:10.1016/s2095-3119(14)60870-4 (2015).

178 Kuşçu, H. *et al.* Economic Return versus Crop Water Productivity of Watermelon under Full and Deficit Irrigation Conditions. *Toprak Su Dergisi* **6**, 7-14 (2017).

179 Yoosefzadeh Najafabadi, M., Soltani, F., Noory, H. & Díaz-Pérez, J. C. Growth, Yield and Enzyme Activity Response of Watermelon Accessions Exposed to Irrigation Water Deficit. *International Journal of Vegetable Science* **24**, 323-337, doi:10.1080/19315260.2017.1419329 (2018).

180 Abdelkhalik, A. *et al.* Yield response of seedless watermelon to different drip irrigation strategies under Mediterranean conditions. *Agricultural Water Management* **212**, 99-110, doi:10.1016/j.agwat.2018.08.044 (2019).

181 Pawar, P. S., Kadale, A. S. & Gadade, G. D. Effect of Various Mulches and Irrigation Levels on Root Zone Temperature, Growth and Fruit Yield of Watermelon (Citrullus lanatus T.) cv. Kiran. *International Journal of Current Microbiology and Applied Sciences* **8**, 2566-2576, doi:10.20546/ijcmas.2019.810.297 (2019).

182 Enyew, A., Tewabe, D., Tsige, A. & Tejada Moral, M. Determining the irrigation regime of watermelon at Koga and Rib irrigation schemes in Amhara Region, Ethiopia. *Cogent Food & Agriculture* **6**, doi:10.1080/23311932.2020.1730108 (2020).

183 khalifa, r. Effect of different irrigation water levels and bio-minerals fertilization on fruit yield, quality and water productivity of watermelon grown on sandy soil, Egypt. *Egyptian Journal of Soil Science* **0**, 0-0, doi:10.21608/ejss.2020.29343.1355 (2020).

184 Qin, K. & Leskovar, D. I. Assessments of Humic Substances Application and Deficit Irrigation in Triploid Watermelon. *HortScience* **55**, 716-721, doi:10.21273/hortsci14872-20 (2020).

185 Yavuz, D. *et al.* How do rootstocks of citron watermelon (Citrullus lanatus var. citroides) affect the yield and quality of watermelon under deficit irrigation? *Agricultural Water Management* **241**, doi:10.1016/j.agwat.2020.106351 (2020).
